# Antifungal mechanisms of the antagonistic bacterium *Bacillus mojavensis* UTF-33 and its potential as a new biopesticide

**DOI:** 10.3389/fmicb.2023.1201624

**Published:** 2023-05-24

**Authors:** Yifan Zhang, Yanmei Yang, Luyi Zhang, Jia Zhang, Zhanmei Zhou, Jinchang Yang, Yu Hu, Xiaoling Gao, Rongjun Chen, Zhengjian Huang, Zhengjun Xu, Lihua Li

**Affiliations:** ^1^Crop Ecophysiology and Cultivation Key Laboratory of Sichuan Province, Chengdu, Sichuan, China; ^2^Maize Research Institute, Sichuan Agricultural University, Chengdu, Sichuan, China; ^3^College of Agronomy, Sichuan Agricultural University, Chengdu, Sichuan, China

**Keywords:** antifungal activity, biopesticide, endophyte, *Magnaporthe oryzae*, lipopeptides

## Abstract

Biological control has gradually become the dominant means of controlling fungal disease over recent years. In this study, an endophytic strain of UTF-33 was isolated from acid mold (*Rumex acetosa* L.) leaves. Based on 16S rDNA gene sequence comparison, and biochemical and physiological characteristics, this strain was formally identified as *Bacillus mojavensis*. *Bacillus mojavensis* UTF-33 was sensitive to most of the antibiotics tested except neomycin. Moreover, the filtrate fermentation solution of *Bacillus mojavensis* UTF-33 had a significant inhibitory effect on the growth of rice blast and was used in field evaluation tests, which reduced the infestation of rice blast effectively. Rice treated with filtrate fermentation broth exhibited multiple defense mechanisms in response, including the enhanced expression of disease process-related genes and transcription factor genes, and significantly upregulated the gene expression of titin, salicylic acid pathway-related genes, and H_2_O_2_ accumulation, in plants; this may directly or indirectly act as an antagonist to pathogenic infestation. Further analysis revealed that the n-butanol crude extract of *Bacillus mojavensis* UTF-33 could retard or even inhibit conidial germination and prevent the formation of adherent cells both *in vitro* and *in vivo*. In addition, the amplification of functional genes for biocontrol using specific primers showed that *Bacillus mojavensis* UTF-33 expresses genes that can direct the synthesis of *bioA*, *bmyB*, *fenB*, *ituD*, *srfAA* and other substances; this information can help us to determine the extraction direction and purification method for inhibitory substances at a later stage. In conclusion, this is the first study to identify *Bacillus mojavensis* as a potential agent for the control of rice diseases; this strain, and its bioactive substances, have the potential to be developed as biopesticides.

## Introduction

Rice (*Oryza sativa* L.) is one of the world’s major food crops and also serves as a staple food for more than half of the world’s population (Zeng et al., 2017; Huang et al., 2021). During its life cycle, rice can be attacked by a variety of microbial pathogens, thus causing huge economic losses to agricultural production while restricting the healthy and stable development of the rice industry. In particular, rice blast has a devastating effect on rice production. Rice blast is a worldwide rice disease caused by the ascomycete fungus (*Magnaporthe oryzae*). The disease affects rice all year round, producing lesions on leaves, leaf necks, culms, culm nodes, spike nodes and spike necks. These lesions cause leaf blast, neck blast, and spike blast and the development of spots. The color and shape of the spots vary depending on environmental conditions and the developmental stage of the plant (Law et al., 2017).

Rice fungus is a filamentous ascomycete fungus that exists in two hybrid types: MAT-1 and MAT-2. This fungus can form sexual substrates called ascospores within 21 days with flask-shaped mycelium carrying a large number of ascospores (Talbot, 2003; Ryder and Talbot, 2015). Rice blast begins when asexual spores land on the surface of rice leaves and attach themselves to the cuticle by releasing an adhesive in the apical spacing of the spores (Hamer et al., 1988). The disease causing process can be divided into the following five steps: conidia formation, budding, attachment cell formation, infestation peg differentiation, mycelial infestation expansion, and disease spot formation (Zhang et al., 2016; Osés-Ruiz et al., 2017; Gupta et al., 2021).

At present, the main control methods for rice blast are divided into three strategies: the selection of resistant rice varieties, chemical control, and biological control. Screening disease-resistant varieties can effectively improve the ability of rice to resist external disturbances, reduce the occurrence of disease, and promote the healthy growth of rice. However, the genetic complexity and diversity of the rice fungus itself leads to differences in its pathogenicity; furthermore, rice can exhibit differences in resistance (Ou, 1980). Furthermore, the fungi responsible for plague are prone to mutation which enables them to overcome the resistance of rice by evolutionary processes (Oerke, 1996). Chemical control is an important means of prevention and control because it is rapid and inexpensive; mizoram, paddy strain, tebuconazole, and tricyclozole are common chemical pesticides (Kunova et al., 2013). However, the annual application of a large number of pesticides has led to an increase in the resistance of rice to plague bacteria. Thus, the resistance of rice is becoming weaker; this, combined with environmental pollution, has caused socio-economic losses, and could eventually lead to the poisoning of humans and animals, and increased mortality.

With the increasing concern for sustainable agricultural development, biopesticides are highly valued for their environmental friendliness, low resistance, low harm to natural enemies of pests, and low risk to human and animal health (Zhang, 2020). Biopesticides involve organisms, or their secondary metabolites, producing antagonistic inhibitory effects on diseases (Samada and Tambunan, 2020). According to their source, biopesticides can be divided into plant-derived pesticides and microbial-derived pesticides. Research has shown that *Bacillus* can secrete a variety of hydrolytic enzymes, including lipase, protease, and amylase, because of its strong resistance to adversity, thus leading to high levels of resistance to temperature, ultraviolet radiation, and other factors (Fravel, 2005). Moreover, this bacterium has a wide spectrum of inhibitory properties, rapid reproduction, low production costs, a good safety profile, and a wide variety of advantageous properties for biological control. The main species of *Bacillus* used in biological control research are *Bacillus subtilis*, *Bacillus thuringiensis*, *Bacillus amyloliq uefaciens*, and *Bacillus polymyxa* (Wang et al., 2019; Roy et al., 2021).

In this study, an endophytic strain, named UTF-33, with strong inhibitory effects on rice fungus, was isolated and identified from the leaves of acid mold. The strain was identified by morphological, biochemical, and physiological characterization, and confirmed by 16S rRNA sequence analysis. The drug sensitivity profile of this bacterium was investigated and the effects of its filtrate fermentation solution on the defense mechanism of rice were investigated. The crude extraction of filtered fermentation broth with n-butanol was tested for its inhibitory effects on conidial germination, attachment cell formation, mycelial growth, cell permeability, and the infestation process of *Fusarium oxysporum*. In addition, the effect of antagonistic bacteria on the biological control of rice blast was evaluated in field trials. The detection of active expression genes provided further directions and methods for subsequent extraction and purification. Collectively, these studies confirmed the potential value of strain UTF-33 for development as a biopesticide.

## Materials and methods

### Isolation of endophytic bacteria from plants

The leaves of fresh and healthy acid mold, clover, hoodia, dong quai, cactus, goosefoot, double-podded cassia, leek, and coronary, were collected and placed in anhydrous ethanol, soaked and disinfected for 2–3 min; the mixture was then poured out and the procedure was repeated 2–3 times. Then, the leaves were placed in 1% sodium hypochlorite solution, soaked for 30 s, rinsed with sterile distilled water, dried and grinded by homogenization. After gradient dilution of 10, 10^2^,10^3^and 10^4^ times with sterile distilled water, single colonies were selected and incubated at 28°C for 24–36 h on coated PDA (potato dextrose agar) plates on a shaking platform. In order to exclude the non-endogenous bacteria adhering to the surface of plant leaves from interfering with the test results, sterile distilled water from the last rinsing of leaves was collected and plated on PDA, and then plates were incubated for 48 h. The bacterial growth on the surface of the plates was observed to determine whether all the non-endogenous bacteria on the surface of plant leaves had been killed.

### Screening of antagonistic bacteria for rice blast

The antifungal activity of endophytic bacteria was investigated using Guy11, a standard strain provided by the Rice Research Institute of Sichuan Agricultural University. First, 5 mL of sterilized fermentation broth was filtered by a 0.22 μm filter and added to 100 mL of cool uncoagulated PDA medium. This was shaken well and used to create a drug-containing plate. Then, we inoculated a rice fungus cake on the drug-containing plate and incubate it in a constant light incubator for 7 days at 28°C with alternating light and dark period. The inhibition rate of each endophytic bacterial filtrate ferment was counted, and the most active endophytic strain was selected as the antagonist UTF-33 as the reference strain for further study. The formula used to calculate inhibition rate was as follows:

Inhibition rate (%) = [1−(diameter of *M. oryzae* in the treatment group – diameter of *M. oryzae* cake in the treatment group)/(diameter of *M. oryzae* in the control group – diameter of *M. oryzae* cake in the control group)] × 100.

### Identification of strain UTF-33

Genomic DNA was extracted from strain UTF-33 with a DNA extraction kit and its 16S rRNA sequence was amplified using universal primers [27F (5-AGAGTTTGATCCTGGCTCAG) and 1492R (5-TACGGCTACCTTGTTACGACGACTT)]. PCR (polymerase chain reaction) was then performed according to previous literature (Reid, 1991). PCR amplicons were sent to Tsingke Biological Technology Company (Chengdu) for sequencing analysis after electrophoretic detection. The 16S rDNA sequences were analyzed by BLAST and a phylogenetic tree was constructed for strain UTF-33 using MEGA 11.1 software.

### Antibiotic susceptibility testing

The paper agar diffusion method was used to detect the sensitivity of UTF-33 to various antibiotics. Fresh endophytes were diluted 100 times and applied evenly on the surface of PDA plates. After uniform coating, drug-sensitive tablets containing different antibiotics were placed in the center of the medium, and the plates were placed in an incubator at a constant temperature of 28°C for 24 h. Finally, susceptibility was determined by measuring the size of the corresponding inhibition circle according to clinical and laboratory standards (Humphries et al., 2021).

### Field trials

A trial was conducted to evaluate the control of UTF-33 filtrate fermentation solution on rice blast in the field at Wenjiang District, Chengdu, Sichuan Province, China (30°680 N, 103°850E). The paddy fields were divided into 1 m^2^ blocks of 15 plots (5 groups of treatments, three replications) with a 40–50 cm interval between each plot. Dewy LTH (Lijiangxintuan) seeds were distributed in the field blocks, covered with film until the rice seedlings grew to 15–20 cm, and were cultivated for approximately 15 days after the film was removed. The leaves of LTH in the field were sprayed with 150 mL of sterile water, LB (Luria-Bertani) medium, carbendazim, UTF-33 bacterial solution, UTF-33 filter fermentation solution, and 1 × 10^5^ CFU/mL fungus conidia suspension, mixed in equal amounts. All of these solutions contained 0.1% Tween-20. After treatment, the rice was left to grow naturally for 5–7 days, and the disease susceptibility of each treatment group was observed and quantified by the random sampling of each leaf intercepted 5 cm for the number of disease spots.

### Defensive gene expression

The dewy LTH seeds were arranged in small pots and placed in a light culture room for 28 days (light culture: 28°C, 16 h; dark culture: 28°C, 8 h) until the three-leaf stage. Then, the plants were sprayed with equal amounts of sterile water, UTF-33 filtered fermentation solution, and 1 × 10^5^ CFU/mL of fungus conidia suspension, followed by leaf collection at 0, 24, 48, 72 and 96 h, respectively (Chen et al., 2021). Total RNA was extracted by the Trizol method and cDNA fragments were amplified with a Primes RT kit. qRT-PCR (quantitative real-time PCR) was performed using BIO-RAD ligation and the expression levels of *OsActin* were used as an internal reference for normalization. The primer sequences of the relevant defense genes and the internal reference genes are shown in Table 1.

**Table 1:** The related defense genes detected in this study and the primers used for fluorescent quantitative PCR.

Gene	Primer sequences (5′ → 3′)
Actin	F: GAGTATGATGAGTCGGGTCCAG
	R: ACACCAACAATCCCAAACAGAG
PR1a	F: GCTACGTGTTTATGCATGTATGG
	R: TCGGATTTATTCTCACCAGCA
PR5	F: GGTACAACGTCGCCATGAGCT
	R: TGGGCAGAAGACGACTCGGTAG
CEBiP	F: CATCGCTCATCATACAAACCA
	R: GGAGATAACAGACATGCTCCAC
NH1	F: AAGCGGTTCAAATCTCAAA
	R: GCCTCCATCGGAAACATA
MAPK6	F: CTCGTACCACCTCAGAAAC
	R: AAATACAGCCCACAGACC
LYP6	F: TGCCCAGGACCACATCAGT
	R: CCAGGGAAGCCCGGAATAT
LYP4	F: GCAACTTGGACCTGTTCTGCG
	R: CCTGGGCATTGAGGCTTGAGT
WRKY53	F: ACGGGCAGAAGCAGGTGAAG
	R: CCCTTGTAGACGATCTGGGTGA
WRKY89	F: GCACCTCACAATGATGGA
	R: GGACAGCCTTGCACTTTA
EREBP	F: GTGTTCGTGTCTGGCTTGG
	R: CACTTGACTTGGGTGCTTTA

### H_2_O_2_ accumulation

LTH leaves were treated as described above and leaves were collected at 0, 24, 48, 72 and 96 h post-treatment, respectively. Then, we weighed 50 mg of DAB (3,3’-Diaminobenzidine Tetrahydrochloride) powder and dissolved this with a small amount of concentrated hydrochloric acid. The volume was then made up to 100 mL with distilled water to 100 mL and the pH was adjusted to 3.8. Then, the leaves were treated for different times in DAB staining solution, placed under vacuum for 30 min, and then placed in the dark at room temperature for 12 h (Daudi and O’Brien, 2012). Next, the staining solution was poured off and the leaves were decolorized with 95% alcohol; during this stage, the alcohol was replaced as many times as necessary until the leaves became transparent. Then, the leaves were examined by stereomicroscopy.

### Conidial germination testing and the formation of attachment cells

Conidia were inoculated onto CM (complete medium) plates with Guy11, incubated at 25°C for 7–11 days with alternating light and dark, and then scraped and filtered through non-woven fabric to remove the impurities left by the mycelium and medium. The filtered spore solution was then centrifuged at 10000 r/min for 2 min; then, the supernatant was removed and the procedure was repeated twice. Next, we added an appropriate amount of sterile distilled water to resuspend the spores, mixed thoroughly, and adjusted the concentration to approximately 1 × 10^5^ CFU/mL with a hemocytometer under a microscope (Li et al., 2017). Then, we added n-butanol crude extract and sterile distilled water to the adjusted concentration of spore solution. Then, we took a clean hydrophobic coverslip and added 50 μL of spore solution so that there were approximately 20 drops of spore solution on one coverslip. The coverslips were then placed in a humid petri dish and incubated at 28°C. At 0, 2, 8, 12, 24, and 48 h of incubation, the cover slips were inverted on the slides and sealed with petroleum jelly to create mounts for the observation of conidia germination and the formation of attached spores under a Zeiss fluorescence microscope.

### The gene detection of active substances in endophytic bacteria

The genomic DNA of the antagonist was used as an amplification template and its genomic DNA sequence. Universal primers were then used to amplify the *sfp*, *ituD*, *fenB*, *bioA*, and *bmyB* genes (Table 2). Following electrophoretic detection, the PCR amplification stock solution was sent to Tsingke Biological Technology Company (Chengdu) for sequencing analysis.

**Table 2:** Primers used for amplification of functional genes relevant to this study.

Gene	Primer sequences (5′ → 3′)	PCR products size (bp)
bioA	F:TTCCACGGCCATTCCTATAC	210
	R:TTTGTCCCCTTATCCTGCAC	
srfAA	F:GAAAGAGCGGCTGCTGAAAC	273
	R:CCCAATATTGCCGCAATGAC	
fenD	F:CCTGCAGAAGGAGAAGTGAAG	293
	R:TGCTCATCGTCTTCCGTTTC	
fenB	F:CTATAGTTTGTTGACGGCTC	1,400
	R:CAGCACTGGTTCTTGTCGCA	
ituC	F:TTCACTTTTGATCTGGCGAT	575
	R:CGTCCGGTACATTTTCAC	
ituD	F:ATGAACAATCTTGCCTTTTTA	1,203
	R:TTATTTTAAAATCCGCAATT	
bmyB	F:TGAAACAAAGGCATATGCTC	395
	R:AAAAATGCATCTGCCGTTCC	

### Statistical analysis

Statistical analysis was performed using IBM SPASS Statistics 24.0 software. All values are presented as the mean ± SD of at least three independent experiments. Statistically significant differences were calculated by Duncan’s multiple range test and Student’s *t*-test with *p* < 0.05.

## Results

### Isolation and screening of endophytic bacteria

**Figure 1:**
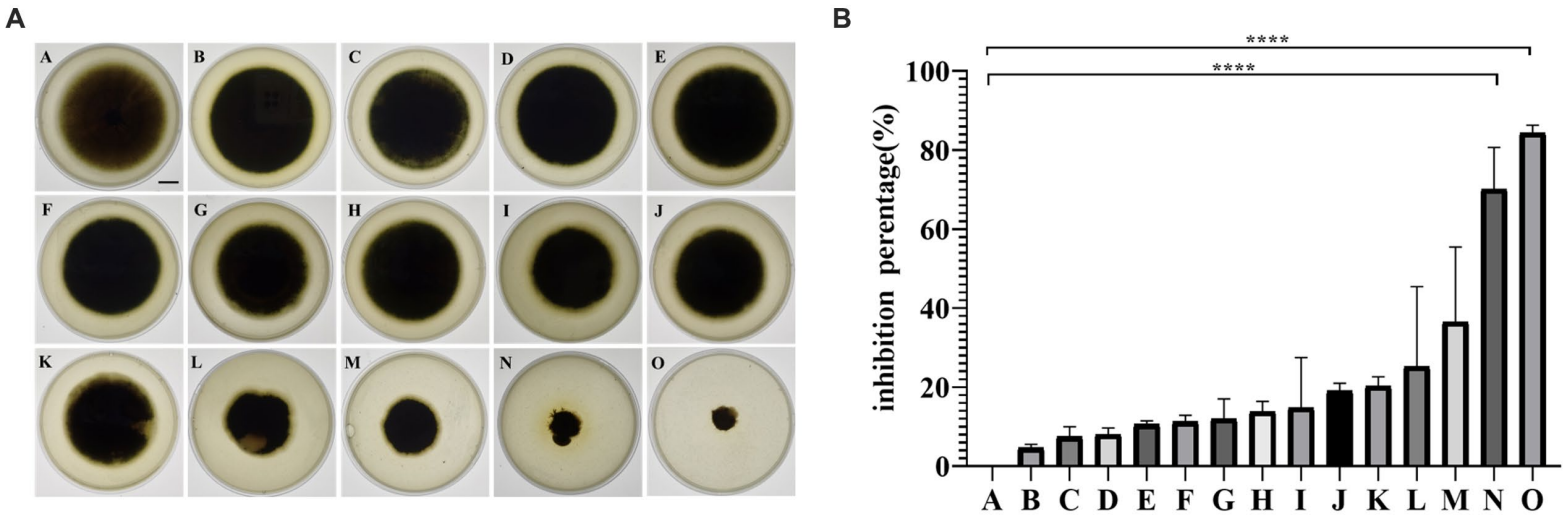
The antifungal activity of isolated endophytes against *Magnaporthe oryzae* **(A)**. A, negative control; B, LHB-Y2-1; C, LH-2; D, HYC-Y2-1; E, HD-6; F, M-11; G, DHC-6; H, DHC-12; I, YC-Y1-1; J, HD-3; K, YC-3; L, SYC-Y1-1; M, SYC-Y3-9; N, SM-Y1-1; O, SM-Y4-3. Scalar bar, 10 mm. The antifungal activity of isolated endophytes against *Magnaporthe oryzae* **(B)**. Significant differences: **** difference is significant at the 0.0001 level.

Fourteen strains (Figure 1A) of endophytic bacteria were isolated from the leaves of fresh sour mold, clover, hoodia, dong quai, cactus, goosefoot, double-pod deciduous, leek and coronary. The antagonistic bacterium SM-Y4-3, which had the highest inhibition rate of 84.35% (Figure 1B), was obtained as the endophytic bacterium of acid mold leaves following the inhibition test; this was referred to as the antagonistic bacterium UTF-33 for further study.

### Morphological characteristics and identification of strain UTF-33

**Figure 2:**
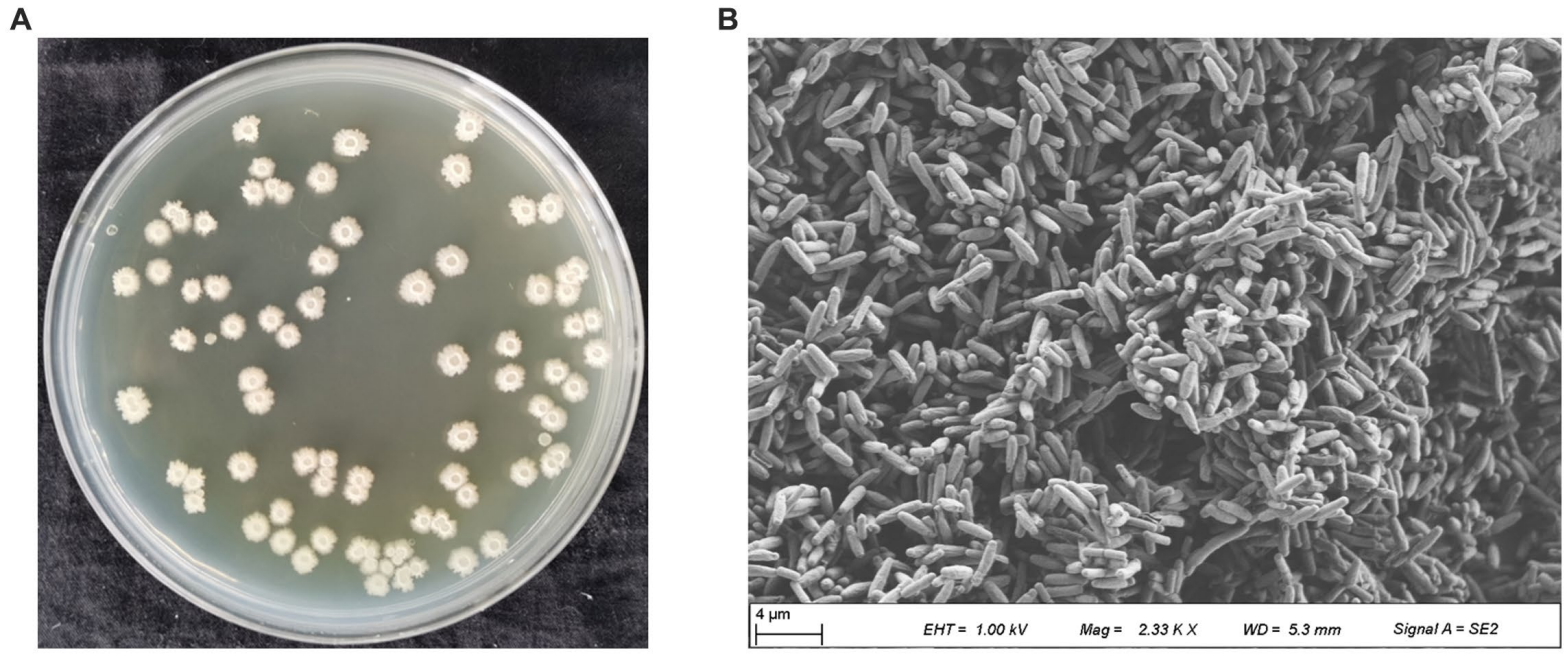
Colony morphology of antagonistic UTF-33. Apparent morphology **(A)**, Microscopic morphology **(B)**.

**Figure 3:**
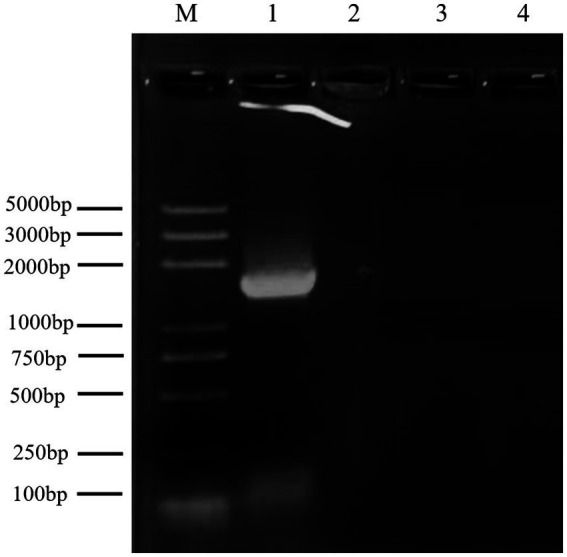
Electrophoretic analysis of UTF-33 16S rDNA. Lane M: Marker 100–5000 bp; Lane 1: UTF-33 16S rDNA.

**Figure 4:**
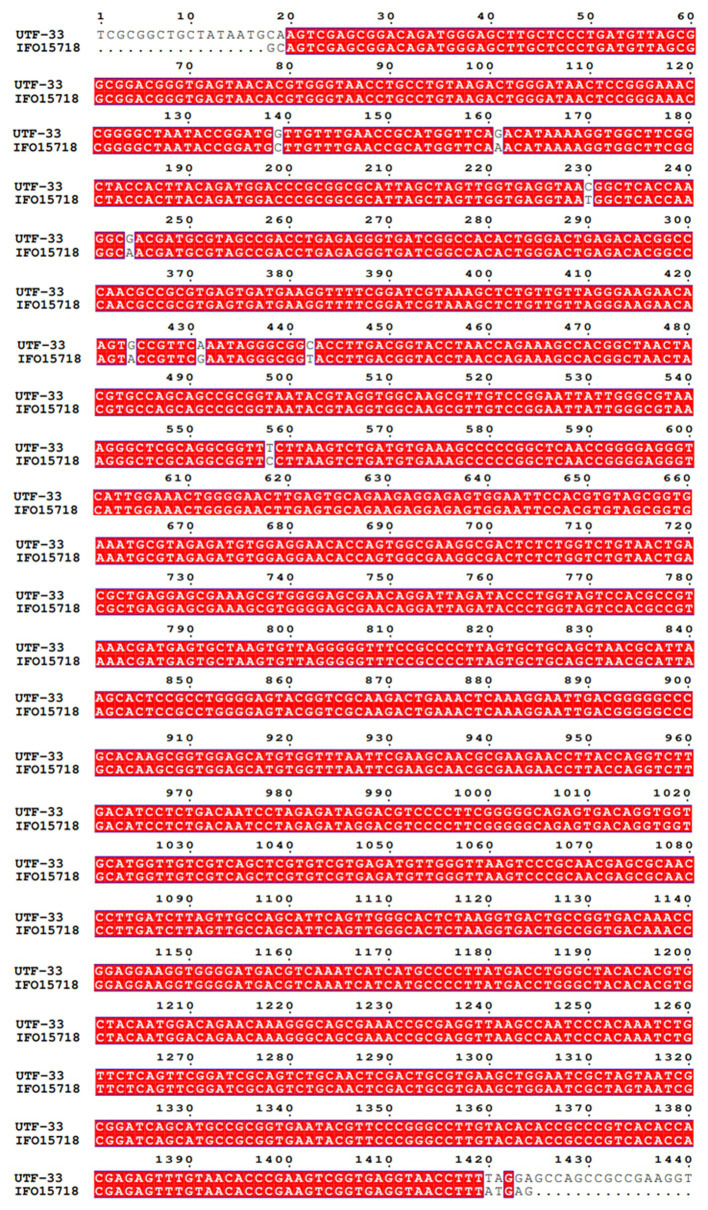
Sequence comparison of 16S rDNA.

**Figure 5:**
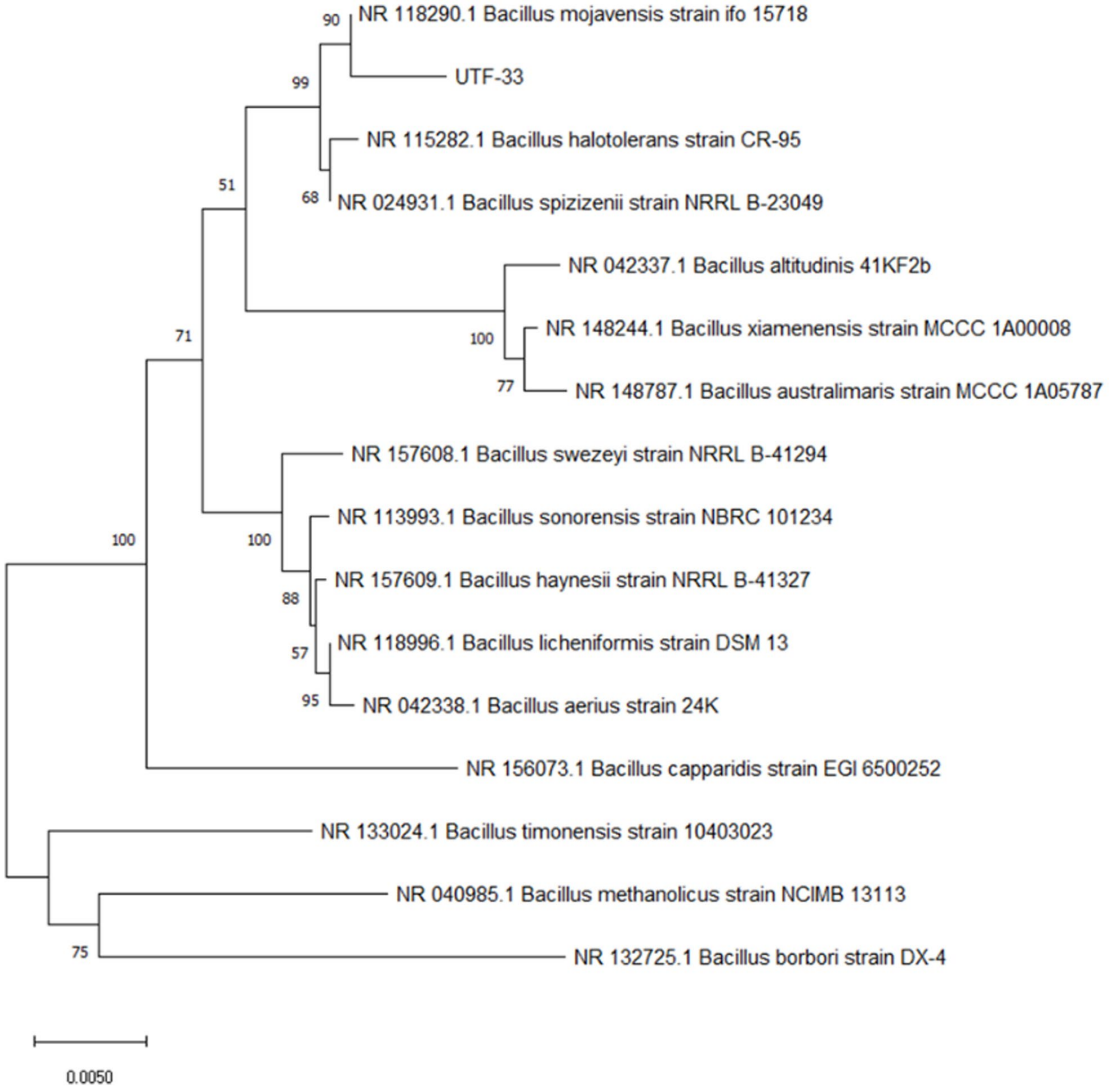
Phylogenetic tree of antagonistic UTF-33.

The antagonistic bacterium UTF-33 grew normally on beef paste peptone medium (light yellow transparent medium), as shown in Figure 2A; colonies were round, milky white and opaque, with folds on the surface and a slight elevation in the middle; the microscopic structure was straight rod with a length of approximately 2–3 microns (Figure 2B). The results of genomic DNA extraction were excellent, with an OD_260_/OD_280_ of 1.83 and a 16S rDNA sequence length of 1,440 bp. The results of electrophoresis are given in Figure 3. Comparative analysis showed that the similarity to *Bacillus mojavensis* strain IFO 15718 was 99.43% (Figure 4); thus, the antagonist UTF-33 was named *Bacillus mojavensis* UTF-33. Figure 5 shows its affinities with other strains.

### Antibiotic susceptibility testing

**Figure 6:**
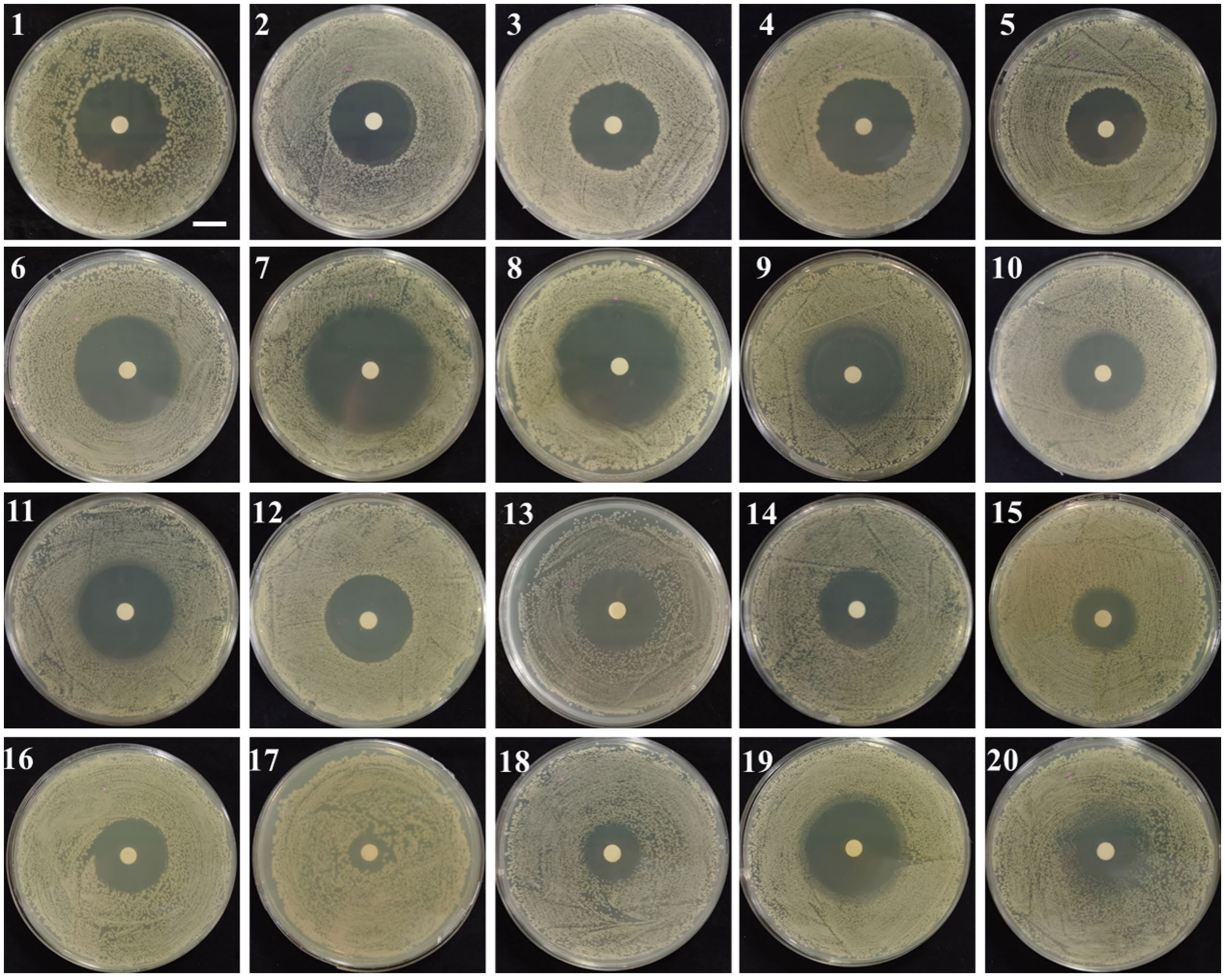
Drug susceptibility testing. 1, Penicillin; 2, oxacillin; 3, Ampicillin; 4, Carboxybenzyl penicillin; 5, piperacillin; 6, Cephalexin; 7, Cefazolin; 8, Cefradine; 9, Cefuroxime; 10, Ceftazidime; 11, Ceftriaxone; 12, Cefoperazone; 13, Polytetracycline; 14, Amikacin; 15, Gentamicin; 16, Kanamycin; 17, neomycin; 18, Tetracycline; 19, minocycline; 20, Erythromycin. Scalar bar, 10 mm.

Analysis (Figure 6) showed that strain UTF-33 was sensitive to most antibiotics, including erythromycin (15 μg), tetracycline (30 μg), polytetracycline (30 μg) and minocycline (30 μg) of macrolides; gentamicin (10 μg), kanamycin (30 μg) and bupropion (30 μg) of aminoglycosides; penicillin (10 μg), ampicillin (10 μg), carbenicillin (100 μg), benzocillin (30 μg) and piperacillin (100 μg) of penicillins; cefazolin (30 μg), cefradine (30 μg), cefadroxil (30 μg), cefoperazone (75 μg), ceftriaxone (30 μg), cefuroxime (30 μg) and ceftazidime (30 μg) of cephalosporins. There were clear circles evident on the culture medium; however, there was resistance to neomycin (30 μg) of aminoglycosides, with no clear circles produced.

### Field trials

**Figure 7:**
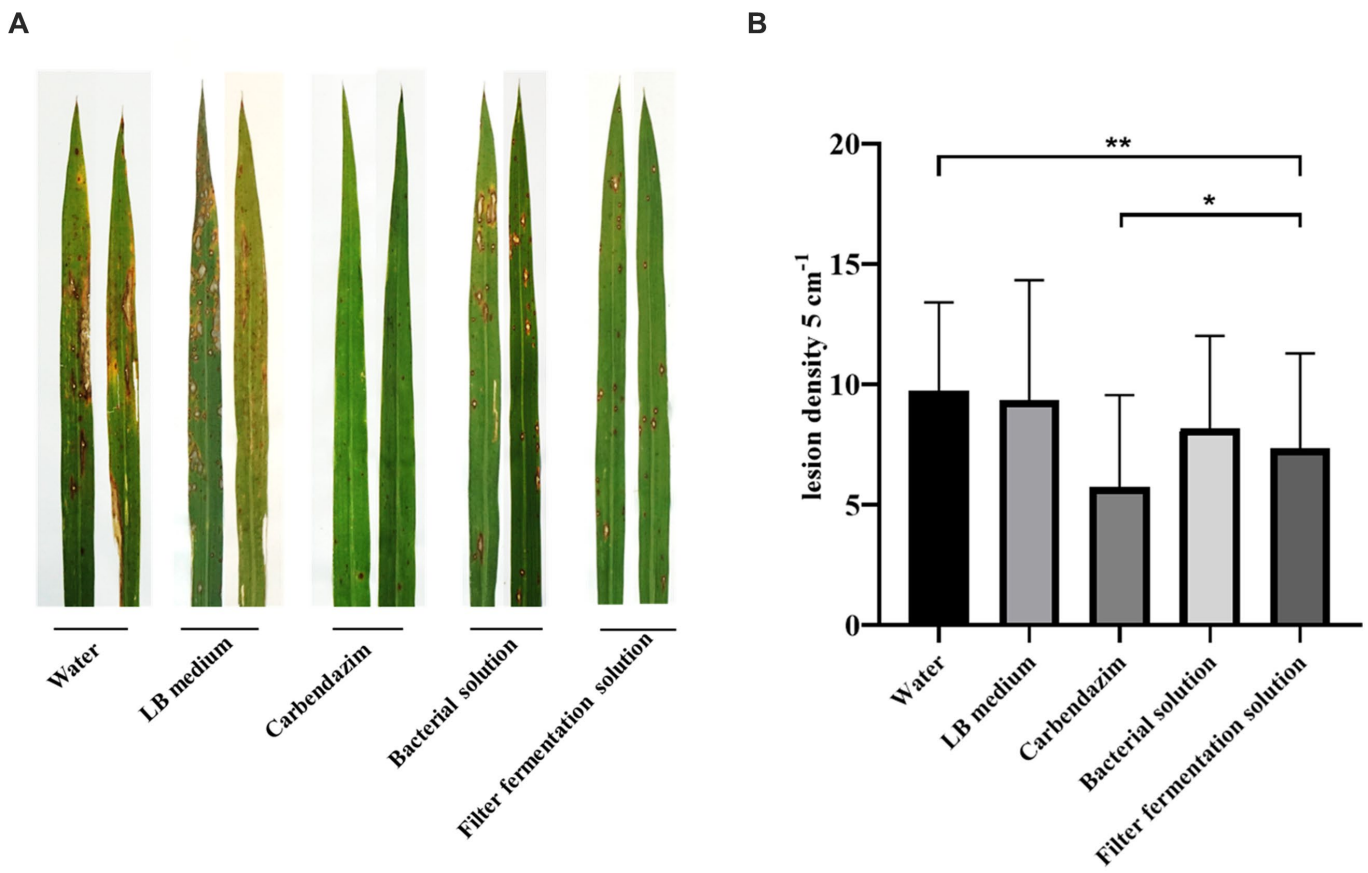
Disease spots of rice blast Guy11 after 7 days of infection with rice after treatment under different conditions **(A)**. Statistical analysis of the number of disease spots after 7 days **(B)**. The number of disease spots in 5 cm per leaf was randomly sampled. Significant differences: * difference is significant at the 0.05 level; **difference is significant at the 0.01 level.

As shown in Figure 7, the incidence of leaf disease and disease spot in the mycorrhizal solution and fermentation filtrate treatment groups were significantly lower than those of the water and LB treatment groups; the difference was smaller than that with the carbendazim treatment group, in which the inhibitory effect of antagonistic bacteria UTF-33 filtrate fermentation solution was more significant.

### Defensive gene expression

The *OsActin* gene was used as an internal reference to detect the normalized expression of genes related to the rice defense response, which was divided into three main categories: (1) genes related to disease course, including *OsPR* family genes such as *OsPR1a*, *OsPR10a* and *OsPR5* (van Loon et al., 2006; Breen et al., 2016); (2) genes related to rice signal transduction, including salicylic acid signaling receptor gene *OsNH1*, titin signaling receptor genes *OsCEBiP*, *OsLYP6*, and *OsMAPK* signaling pathway genes (Yuan et al., 2007); (3) genes encoding transcription factors, including genes from the *OsEREBP* and *OsWRKY* families (Pandey and Somssich, 2009; Birkenbihl et al., 2017).

**Figure 8:**
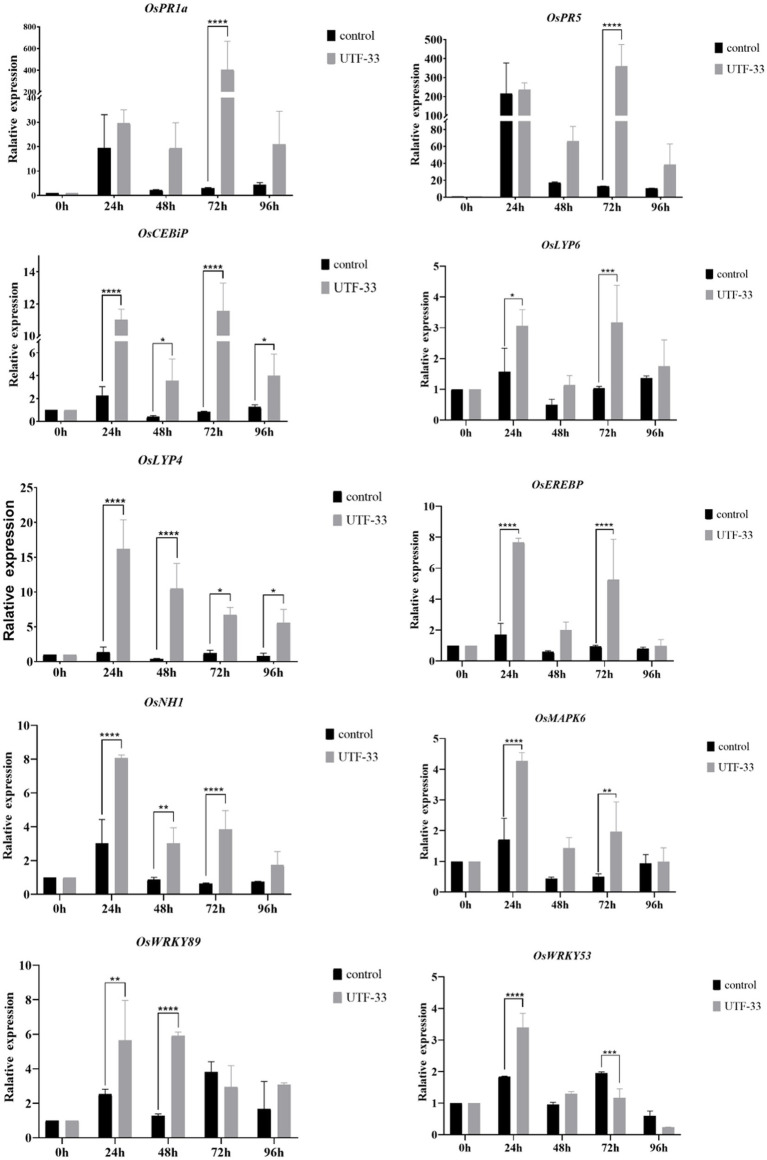
Differences in the expression of antagonistic UTF-33 defense genes in rice. Significant differences: * difference is significant at the 0.05 level; **difference is significant at the 0.01 level; *** difference is significant at the 0.001 level; **** difference is significant at the 0.0001 level.

Analysis (Figure 8) indicated that all rice-related defense genes were differentially upregulated in response to treatment. The expression of genes related to disease process, *OsPR1a* and *OsPR5* genes, peaked at 72 h. Compared with the control group, the expression of *OsPR1a* was upregulated by approximately 90-fold while that of *OsPR5* was upregulated by approximately 30-fold. The expression of *OsCEBiP* and *OsLYP6* peaked at 72 h while that of *OsLYP4* peaked at 24 h. Compared with the control group, the expression of *OsCEBiP* was up-regulated by approximately 11-fold, *OsLYP6* by approximately 3-fold, and *OsLYP4* by approximately 15-fold. The expression levels of both the salicylate signaling receptor gene *OsNH1* and the *OsMAPK6* signaling pathway gene peaked at 24 h. Compared with the control group, the expression of *OsNH1* was up-regulated by approximately 3-fold while that of *OsMAPK6* was up-regulated by approximately 4-fold. The expression of genes encoding transcription factors (*OsEREBP*, *OsWRKY53* and *OsWRKY89*) peaked at 24 h (the expression of *OsWRKY89* at 24 and 48 h was very similar), while the expression levels of *OsEREBP* were up-regulated by approximately 7-fold, *OsWRKY53* by approximately 2-fold, and *OsWRKY89* by approximately 6-fold when compared with the control group. In summary, the antagonistic bacterium UTF-33 promoted the expression of rice defense genes, and the concentrated response was expressed in the early and mid-late stages after rice treatment.

### H_2_O_2_ accumulation

**Figure 9:**
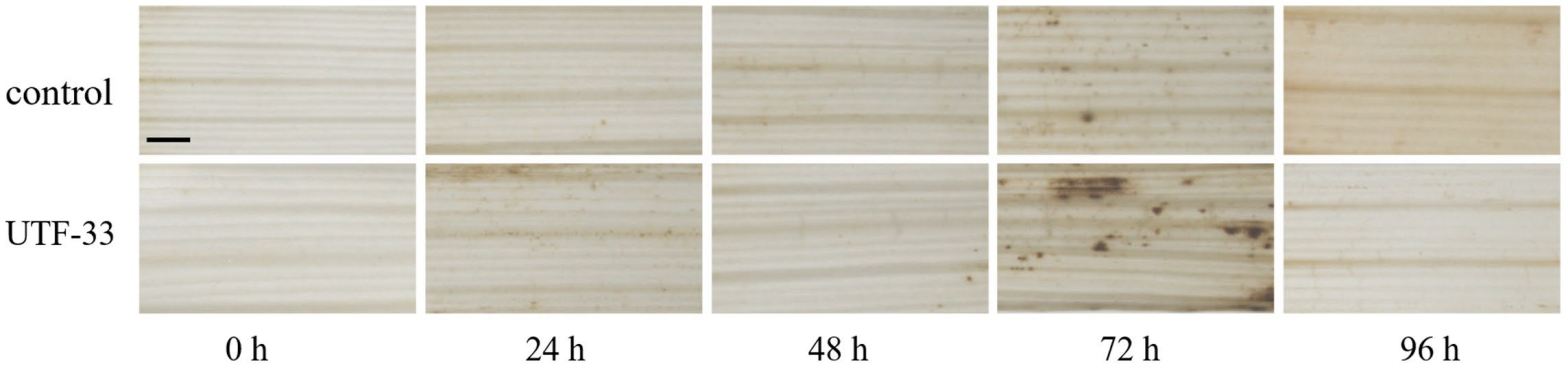
Differences in H_2_O_2_ accumulation arising from antagonistic UTF-33 in rice leaves. Scale bar, 50 μm.

Analysis (Figure 9) showed that the control group showed the deposition of some brown at both 24 and 72 h, thus indicating the presence of H_2_O_2_ accumulation (as detected by DAB staining); the most intense DAB staining was observed in leaf material at 72 h, thus showing the maximal accumulation of H_2_O_2_.

### Conidial germination testing and the formation of attachment cells

**Figure 10:**
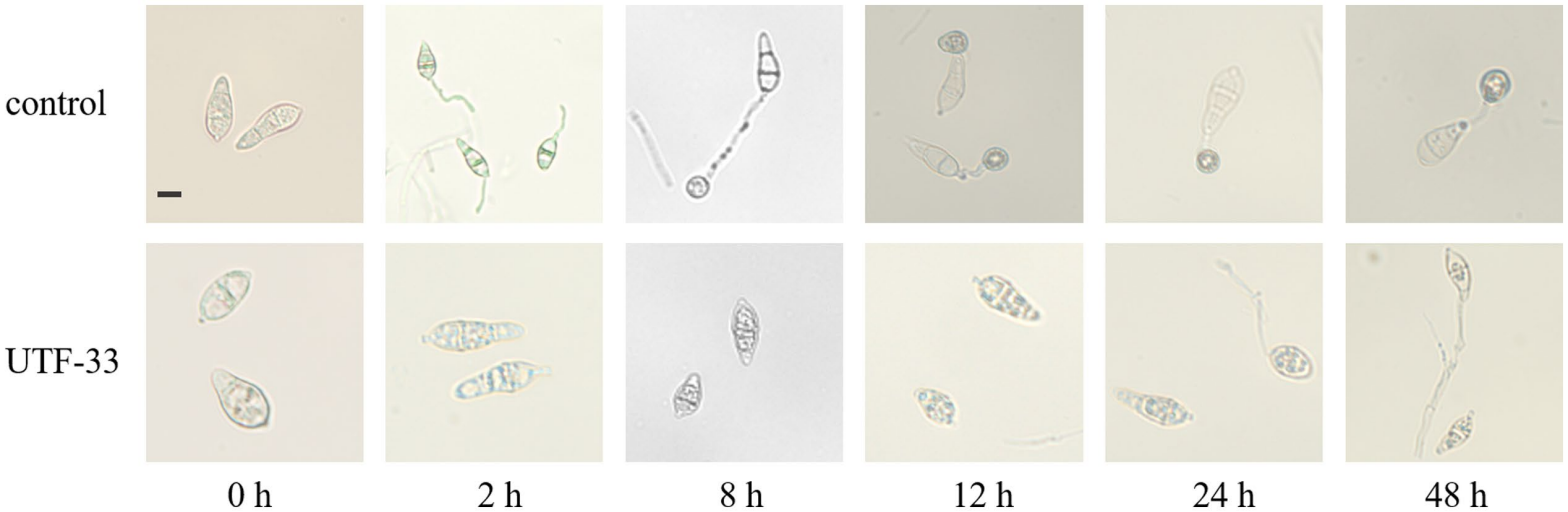
Effect of n-butanol crude extract on conidial germination and the formation of appressorium. Scale bar, 10 μm.

As shown in Figure 10, conidia in the blank group started to generate budding tubes after 2 h; the germination rate was 75.62% and no attached cells were formed. At 8 h, the budding tubes kept elongating, the germination rate was 83.11%, and the attached cell formation rate was 71.79%. At 12 h, melanin deposition was clearly evident in the attached cells, and the germination rate and attached cell formation rate were 86.81 and 76.12%, respectively. At 24 and 48 h, the bud tube germination rate in the blank group increased to 87.8 and 92.98%, and the attached cell formation rate was 78.64 and 72.89%. In the treatment group, a very small number of spores were found to have germinated from 8 h; the germination rate remained below 10% until 48 h and some of the conidia were found to be distorted at 24 h and 48 h. Furthermore, the germinated budding tubes were swollen and broken; no normal attached cell structures were produced throughout the entire experiment.

### The detection of genes associated with active substances in endophytic bacteria

**Figure 11:**
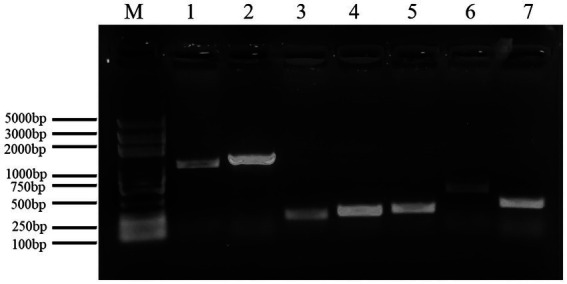
Electrophoresis analysis of functional gene amplification fragments. Lane M: Marker 100–5000 bp; Lane 1–7 is corresponding to gene ituD, fenB, bioA, fenD, srfAA, ituC, bmyB, respectively.

**Figure 12:**
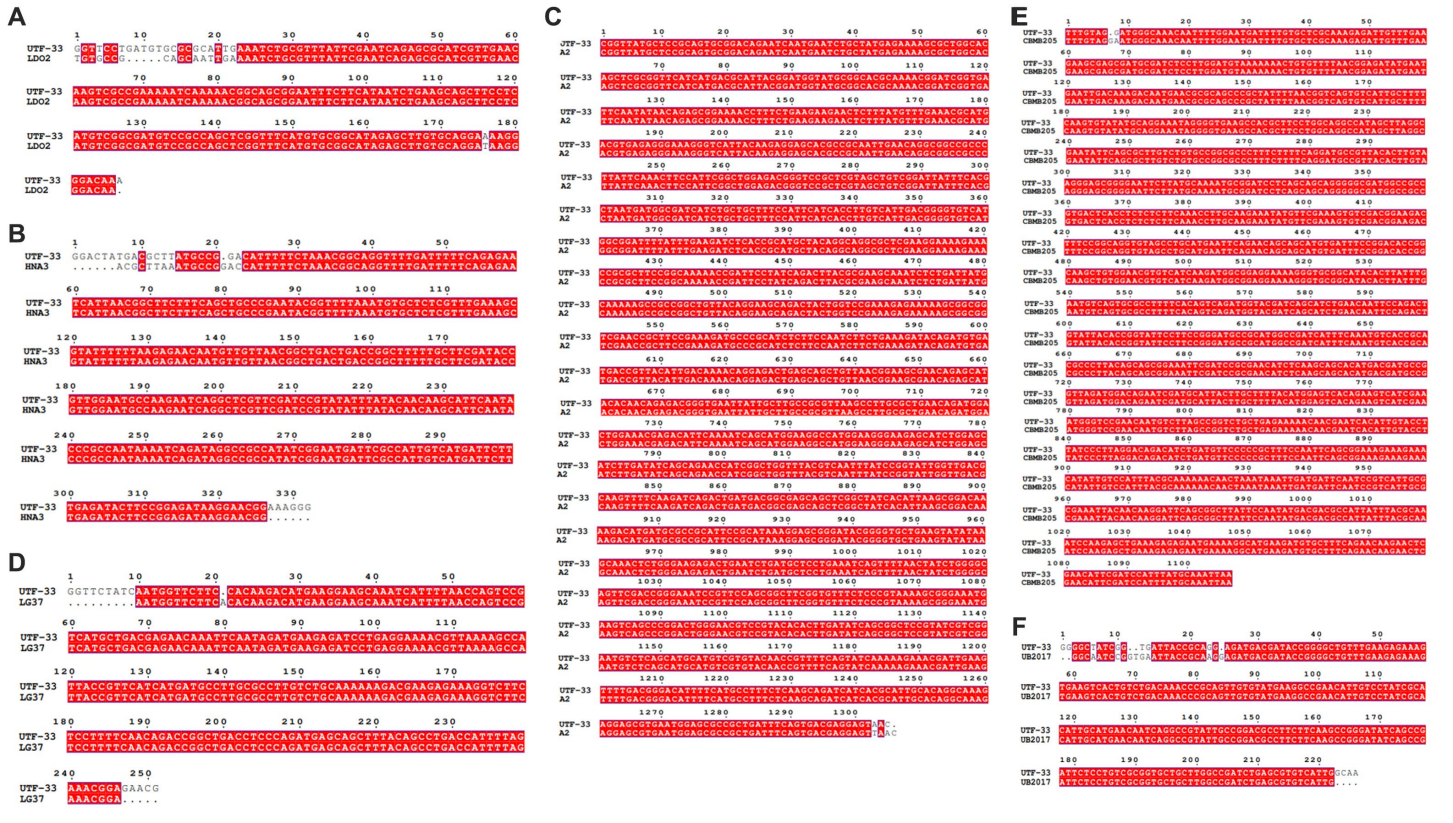
Nucleic acid sequence comparison plots of functional genes. Panels **(A–F)** are corresponding to gene *bioA*, *bmyB*, *fenB*, *fenD*, *ituD* and *srfAA*, respectively. LDO2: *Bacillus velezensis* LDO2, HNA3: *Bacillus sp*. HNA3, A2: *Bacillus velezensis* A2, LG37: *Bacillus velezensis* LG37, CBMB205: *Bacillus velezensis* CBMB205, UB2017: *Bacillus velezensis* UB2017.

**Figure 13:**
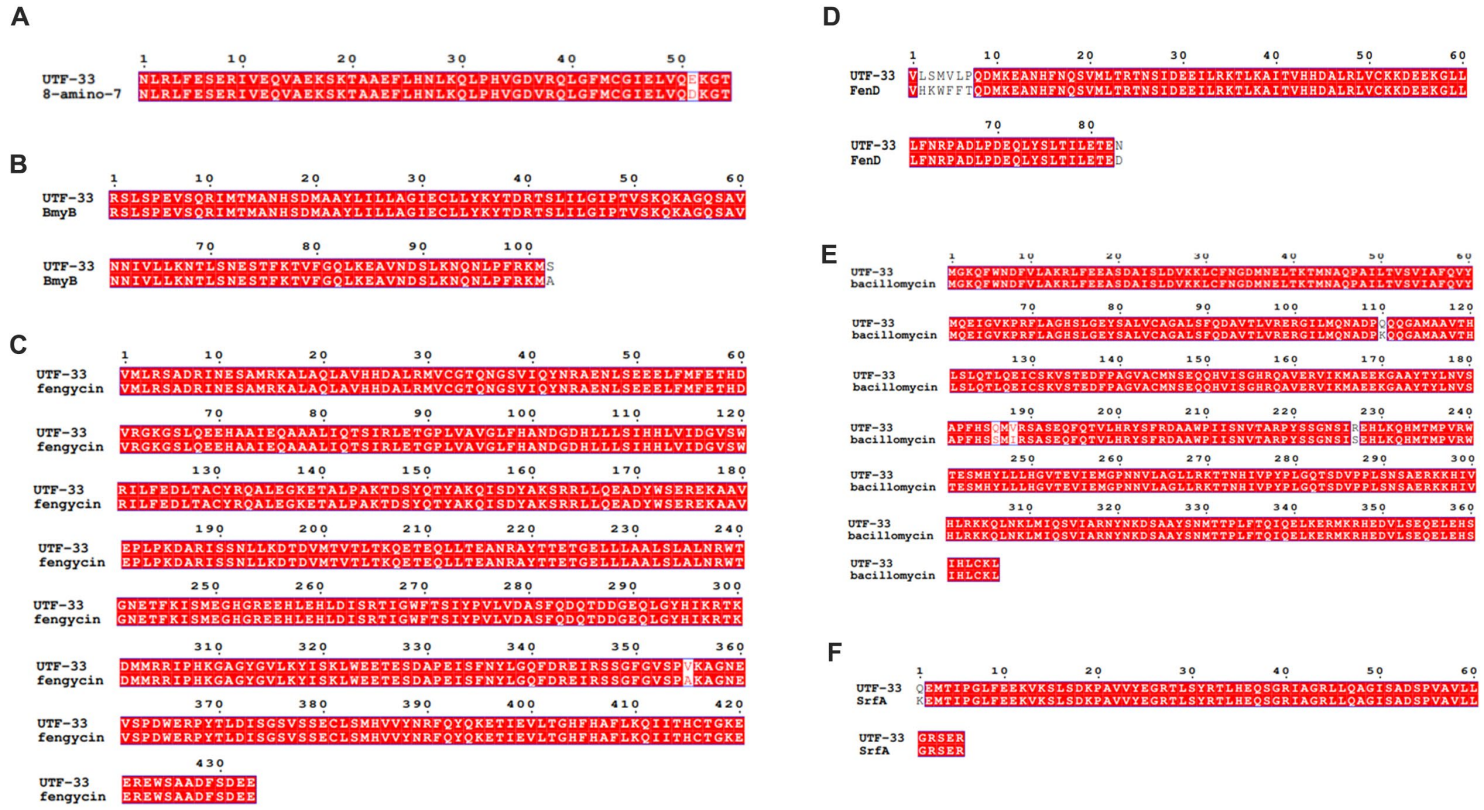
Comparative amino acid sequences of functional genes. Panels **(A–F)** are corresponding to amino acid sequence comparisons of gene *bioA*, *bmyB*, *fenB*, *fenD*, *ituD* and *srfAA*, respectively. 8-amino-7: adenosylmethionine--8-amino-7-oxononanoate transaminase in *Bacillus velezensis*, BmyB: bmyB in *Bacillus subtilis*, fengycin: fengycin synthetase in *Bacillus subtilis*, FenD: FenD in *Bacillus subtilis*, bacillomycin: bacillomycin D biosynthesis malonyl-CoA transacylase BamD in *Bacillus velezensis*, SrfA: surfactin non-ribosomal peptide synthetase SrfAA in *Bacillus velezensis*.

Figure 11 shows a functional gene amplification fragment electrophoresis map. The gene fragment sizes of *bioA*, *bmyB*, *fenB*, *fenD*, *ituD* and *srfAA* obtained after sequencing analysis were 187 bp, 332 bp, 1,306 bp, 252 bp, 1,108 bp and 187 bp, respectively, corresponding to the number of encoded amino acid residues of 54, 156, 433, 83, 366 and 65, respectively. The nucleic acid sequences of each gene were compared by NCBI; analysis showed that these functional genes had very high and uniform similarity with *Bacillus velezensis*, *Bacillus* sp. and *Bacillus amyloliquefaciens* strains featuring corresponding genes, with 97.24, 99.07, 99.92, 99.58, 99.91 and 98.21% similarity, respectively. The nucleic acid sequence comparison with common strains is shown in Figure 12. The results of amino acid sequence comparison matched with the nucleic acid comparison, but since the expression of genes in different strains differed to some extent, the strains with the highest similarity and their corresponding proteins were selected to generate sequence comparison maps (Figure 13). The *bioA*, *bmyB*, *fenB*, *fenD*, *ituD* and *srfAA* genes corresponded to adenosylmethionine - 8-amino-7-oxononanoate transaminase in *Bacillus velezensis*, bmyB in *Bacillus subtilis*, fengycin synthetase in *Bacillus subtilis*, FenD in *Bacillus subtilis*, bacillomycin D biosynthesis malonyl-CoA transacylase BamD in *Bacillus velezensis*, and surfactin non-ribosomal peptide synthetase SrfAA in *Bacillus velezensis*, with similarities of 98.15, 99.02, 99.77, 91.57, 98.91 and 97.06%, respectively. In summary, the functional genes involved in the synthesis of lipopeptides were successfully detected in the antagonist bacterium UTF-33.

## Discussion

As the global population increases, agriculture and natural resources are facing unprecedented demands. In order to meet the needs of the world’s future food security and sustainable development, there is a need to increase food production while effectively reducing environmental hazards (Foley et al., 2011; Zeng et al., 2017). To alleviate the problem of rice yield reduction caused by rice plague, many chemicals have been used as pesticides and fungicides over recent years. However, many of these have been gradually replaced due to the environmental pollution and drug residues they cause; thus increasing interest in the use of biopesticides. Microbial pesticide control effect, production of raw materials and active ingredients are natural products, safe, non-toxic residue-free, to ensure sustainable development (Starnes et al., 1993; Fravel, 2005). *Bacillus*, as one of the most important microbial pesticides, exerts inhibitory effects through mechanisms such as competitive action, antagonism, and the induction of plant resistance (Bacon et al., 2001; Durairaj et al., 2017; Hasan et al., 2020; Diabankana et al., 2021).

In this study, an endophytic strain of the antagonistic bacterium UTF-33 was extracted from the plant acid mold, a perennial herb that is rich in vitamin A, vitamin C, and oxalic acid; this herb is often used as a seasoning for cooking. The plant itself is edible, and it can be inferred that its endophytic bacteria have no toxic effects on human body. The antagonistic bacteria UTF-33 was identified as *Bacillus mojavensis* UTF-33 by physiological and biochemical indicators and by molecular biology. A review of published information on *Bacillus mojavensis* species showed that this bacterium is non-toxic and non-pathogenic; thus, the endophyte can be guaranteed in terms of crop safety and suitable to be developed as biopesticides (Fanaei, 2018; Camele et al., 2019; Mnif et al., 2021). Further analysis found that *Bacillus mojavensis* UTF-33 was sensitive to most of the antibiotics tested, thus indicating that it was not a drug-resistant microorganism, a characteristic consistent with the use of biocontrol bacteria.

The plant’s innate immune system consists of two immune responses: PTI (PAMP-triggered immunity) and ETI (effector-triggered immunity) (Li et al., 2015). PTI is mainly induced by the stimulation of pathogen-associated molecular patterns on the surface of pathogenic microorganisms, thus leading to non-specific defense responses in plants, including the production of reactive oxygen species ROS, *MAPK* activation, and SA (salicylic acid) production. ETI is triggered by the recognition of effector proteins produced by pathogenic microorganisms by the plant’s disease-resistant R proteins, including the production of resistant SAR (systemic acquired resistance) and the activation of various protein kinase cascades (Feys and Parker, 2000). The defense mechanism in rice is specifically divided into three expression pathways: those related to disease course, those related to signal transduction, and those related to the encoding of transcription factors (Yuan et al., 2007; Pandey and Somssich, 2009; Mahdavi et al., 2012; Liu et al., 2016).

After treatment with the fermentation products of *Bacillus mojavensis* UTF-33, *PR1a* and *PR5* (genes related to the course of disease) reached maximal expression levels at 72 h. The genes associated with the titin signaling receptors (*OsCEBiP* and *OsLYP4* and *OsLYP6*) also reached maximal levels during the same period. *OsCEBiP* is known to play a key role in the perception (Kaku et al., 2006) and transduction of titin oligosaccharide inducers and *OsLYP4* and *OsLYP6* encoding proteins that are capable of sensing PGN (peptidoglycan) and fungal titin as bifunctional PRs (pathogenesis-related proteins) (Liu et al., 2012). The genes encoding the *OsEREBP*, *OsWRKY53* and *OsWRKY89* transcription factors were upregulated significantly at 24 h. The expression of the salicylic acid signaling receptor pathway and the *OsMAPK6* signaling pathway (*OsNH1*) also reached maximal levels at this time; this is consistent with the timing and characteristics of the rice autoimmune response when analyzed together with the H_2_O_2_ accumulation results. It was evident that the immune response of the treated rice was concentrated in the early and mid-late stages, with the initial defense against infestation in the early stage; this was due to pathogenic infection stimulating PRS and up-regulating the expression of the fungal titin recognition pathway. The mid-late stage mainly targeted the formation of the infestation pegs, mediating the internal synthesis of SA, and upregulating the expression in concert with multiple transcription factor-related pathways to complete the immune response. In recent studies (Chaiharn et al., 2020; Rong et al., 2020; de Sousa et al., 2021) identified *Bacillus safensis*, *Streptomyces* spp. and *Trichoderma* sp.as potentially being useful for the control of rice blast. These studies mainly focused on the antagonistic effects of microbial pesticides and explored the direct inhibitory effects of metabolites on rice blast. However, we do not know whether these pesticides enhance the autoimmune capacity of rice or activate the response of relevant immune mechanism pathways. However, *Bacillus mojavensis* UTF-33 can indirectly fight against rice fungus infestation by promoting the upregulation of the defense mechanism response in rice itself.

In addition, fermentation products were extracted with n-butanol to obtain crude extracts. We found that these crude extracts had a significant effect in delaying and inhibiting the germination of rice fever conidia and the formation of attachment cells; furthermore, some of the treated conidia showed distortion after 12 h. Most of the mycelium that could germinate also swelled and broke and could not form a normal attachment cell structure. This indicates that these extracts can directly inhibit the developmental growth process of rice fever conidia, as described previously by Chen et al. who treated conidia using the fermentation product of *Bacillus velezensis* (Chen et al., 2021); however, the inhibitory effect of *Bacillus mojavensis* UTF-33 was clearly more effective. Considering both the control effect of biopesticides and the economic effect at the same cost, *Bacillus mojavensis* UTF-33 is more advantageous.

Functional gene amplification experiments were also conducted; we found that *Bacillus mojavensis* UTF-33 expressed genes required for the synthesis of various lipopeptides, including *bioA*, *bmyB* and *fenB*, *ituD*, *srfAA*. *fenB*, *ituD*, and *sfp.* Synthesis products are useful as *Bacillus* extracts are often used in antifungal studies and have recognized potential for use in biocontrol (Zhang et al., 2017; Li et al., 2021; Rasiya and Sebastian, 2021). For example, *B. velezensis* 9D-6 produces surfactins C14 and C15 to antagonize fungal pathogens such as *Fusarium oxysporum*, *Gibberella zeae*, *Pyrenochaeta terrestris* (Grady et al., 2019) and *Bacillus subtilis* WL-2 antagonizes potato blight by producing Iturin A (Wang et al., 2020). In a previous study, Xiao and Hanif purified fengycin from *Bacillus subtilis* Z-14 and *Bacillus amyloliquefaciens* FZB42 to destroy the structure of *Gaeumannomyces graminis* var. *tritici*, and *Fusarium graminearum* to achieve a suppressive effect (Hanif et al., 2019; Xiao et al., 2021). In contrast, *Bacillus mojavensis* UTF-33 has the potential to synthesize a variety of lipopeptides and is presumed to have direct antagonistic effects on a variety of plant pathogens. It is necessary to further purify and investigate the metabolic activity of a variety of antibacterial active substances to investigate their mechanisms of action and broaden the scope of application.

In the field trials, the control effect of *Bacillus mojavensis* UTF-33 as a biopesticide was further evaluated. Leaf disease and spot statistics of the fermented filtrate spray treatment were significantly lower than those of the water and LB treatment groups, and were similar to those of the carbendazim treatment group. Analysis showed that *Bacillus mojavensis* UTF-33 also exhibited excellent bacterial inhibition activity in practical use and could be a beneficial tool for controlling rice blast in the field.

In recent years, Snook et al., Bacon et al., and Hinton et al. used maize as experimental material to study the antagonistic mechanism of *Bacillus mojavensis* against *Fusarium verticillioides*. They also used the metabolites of this species to reduce the lesions of maize stalk seedlings, and developed it as a biopesticide for disease control in maize (Snook et al., 2009; Bacon and Hinton, 2011a,b). Hanen et al., Ayed et al., and Samiha et al. used *Bacillus mojavensis* to extract lipopeptides such as surface activator and eugenol (Ayed et al., 2014; Hanen et al., 2014; Samiha et al., 2014), and also purified the protease synthesized by their metabolism (Beg and Gupta, 2003), or optimizing the production process (Beg et al., 2004; Sepahy et al., 2011). The present literature relating to the biocontrol of crops is relatively homogeneous. This is the first study focusing on the antagonistic effect of *Bacillus mojavensis* on rice diseases. Our findings illustrate the direct inhibitory effect of *Bacillus mojavensis* UTF-33 on rice blast and the indirect induction of the rice defense gene expression mechanism. These data confirm the potential of *Bacillus mojavensis* as a biopesticide and advance the progress of research on the disease suppression mechanisms in plant endophytes.

## Data availability statement

The original contributions presented in the study are included in the article/Supplementary material, further inquiries can be directed to the corresponding authors.

## Author contributions

ZX, RC, YZ, and YY contributed to conception and design of the study. YZ, YY, LZ, and ZZ organized the database. YZ, JZ, XG, and JY performed the statistical analysis. YZ wrote the first draft of the manuscript. YY, YH, ZH, and LL wrote sections of the manuscript. All authors contributed to manuscript revision, read, and approved the submitted version.

## Conflict of interest

The authors declare that the research was conducted in the absence of any commercial or financial relationships that could be construed as a potential conflict of interest.

## Publisher’s note

All claims expressed in this article are solely those of the authors and do not necessarily represent those of their affiliated organizations, or those of the publisher, the editors and the reviewers. Any product that may be evaluated in this article, or claim that may be made by its manufacturer, is not guaranteed or endorsed by the publisher.
