# Supplementary material for: Antifungal mechanisms of the antagonistic bacterium Bacillus mojavensis UTF-33 and its potential as a new biopesticide
Source: Front Microbiol. 2023 May 24;14:1201624. doi: 10.3389/fmicb.2023.1201624 (PMC10246745; doi:10.3389/fmicb.2023.1201624)
Supplement: Supplementary file 1 [file Data_Sheet_1.docx]

**
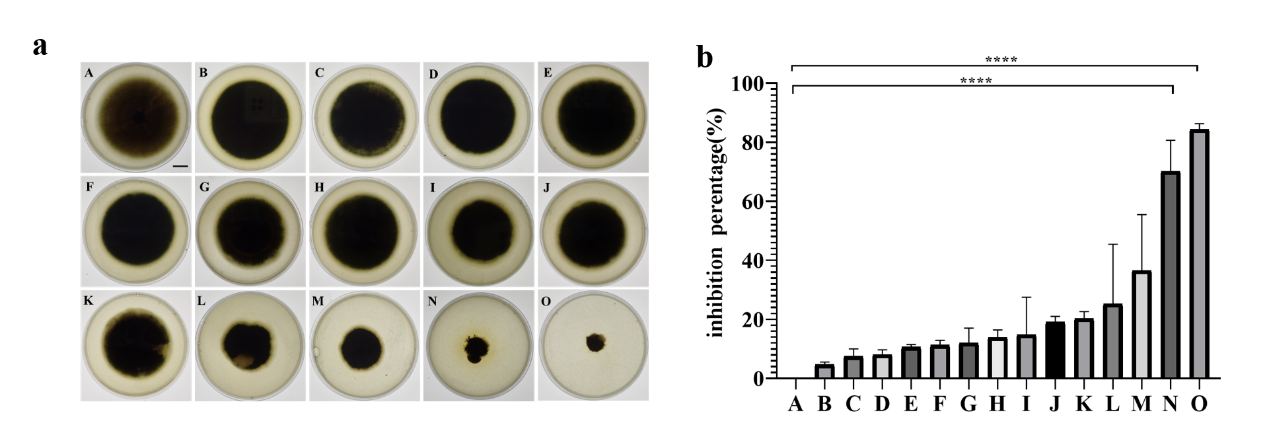
**

**Figure 1.** **The antifungal activity of isolated endophytes against *Magnaporthe oryzae* (a).** A，negative control； B，LHB-Y2-1； C，LH-2； D，HYC-Y2-1； E，HD-6；F，JM-11；G，DHC-6；H，DHC-12；I，YC-Y1-1；J，HD-3；K，YC-3；L，SYC-Y1-1；M，SYC-Y3-9；N，SM-Y1-1；O，SM-Y4-3.Scalar bar, 10 mm. **The antifungal activity of isolated endophytes against *Magnaporthe oryzae* (b).** P<0.01 represents a significant difference.


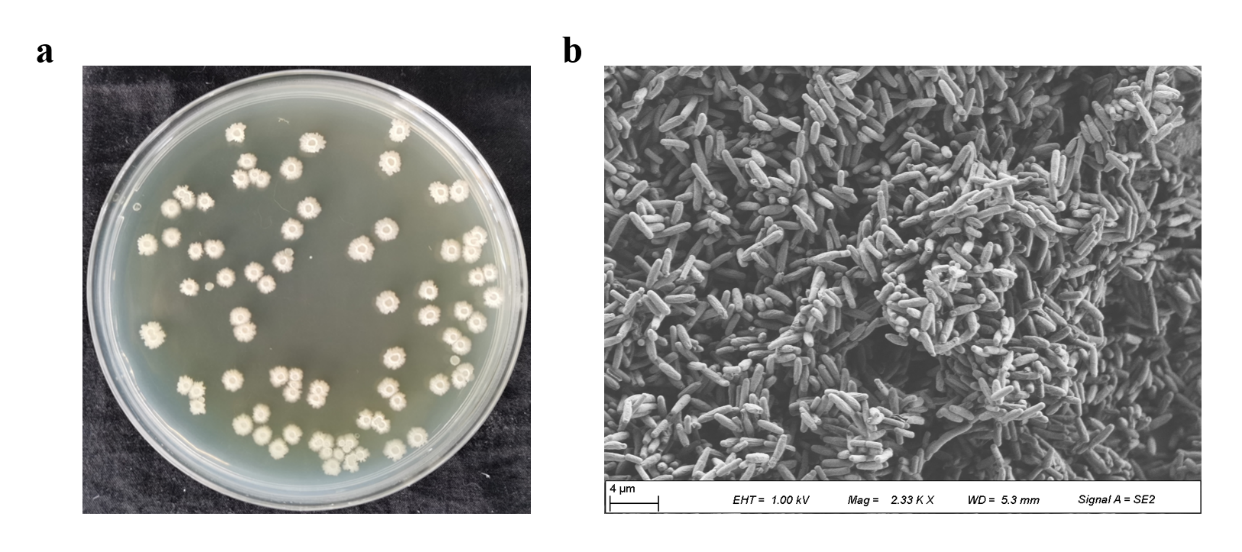


**Figure 2. Colony morphology of antagonistic UTF-33.** **Apparent morphology(a),** **Microscopic morphology(b).**

**
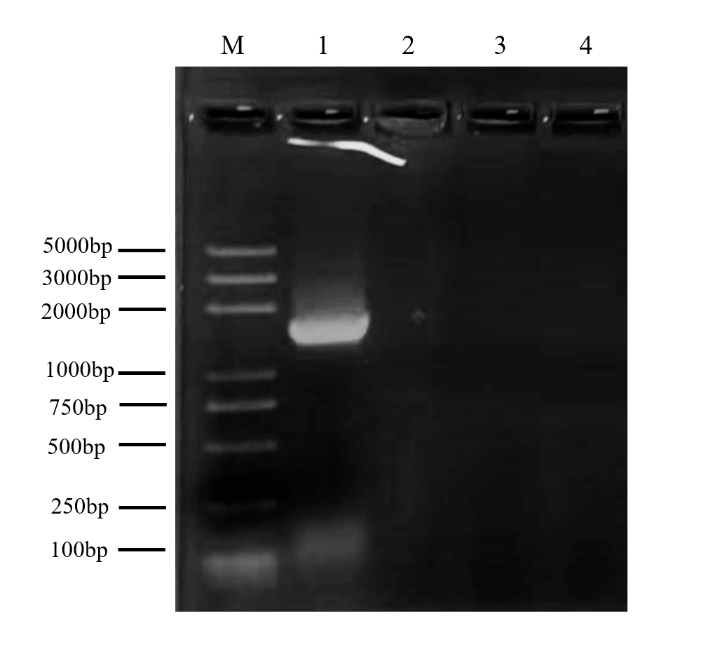
**

**Figure 3. Electrophoretic analysis of UTF-33 16S rDNA**

**
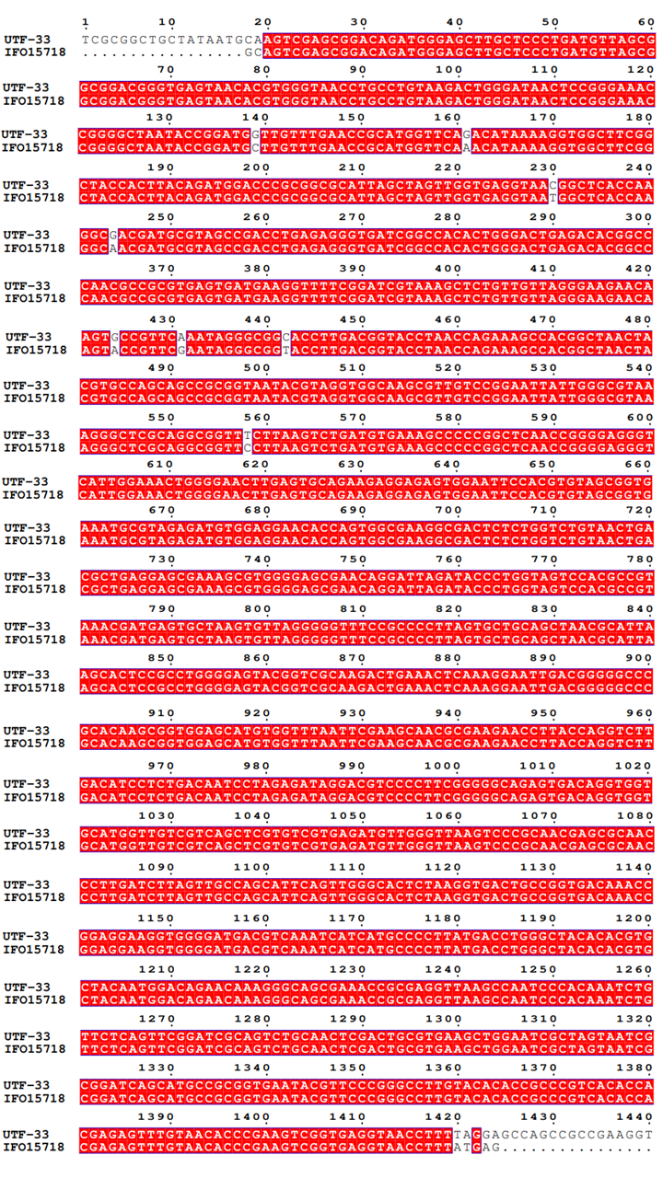
**

**Figure 4. Sequence Comparison of 16S rDNA**

**
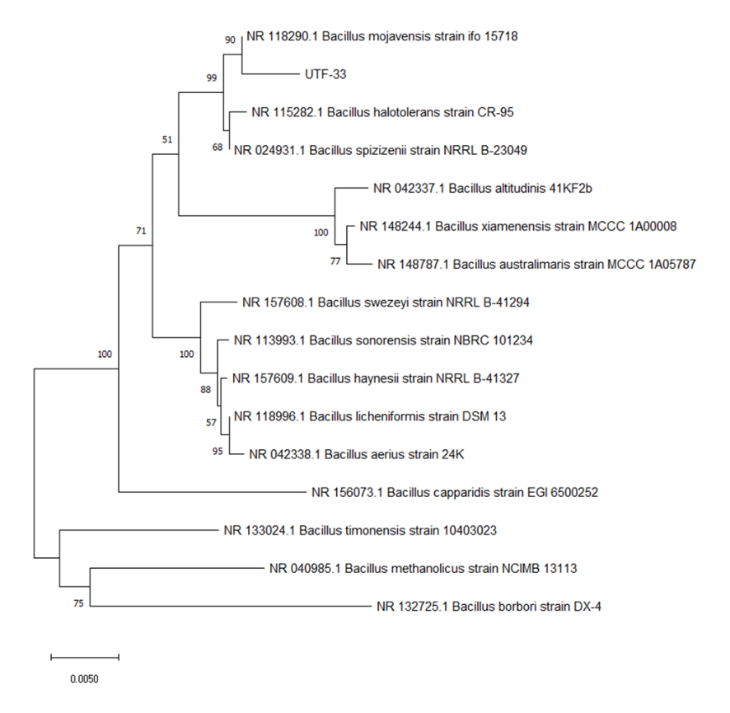
**

**Figure 5. Phylogenetic tree of antagonistic UTF-33**

**
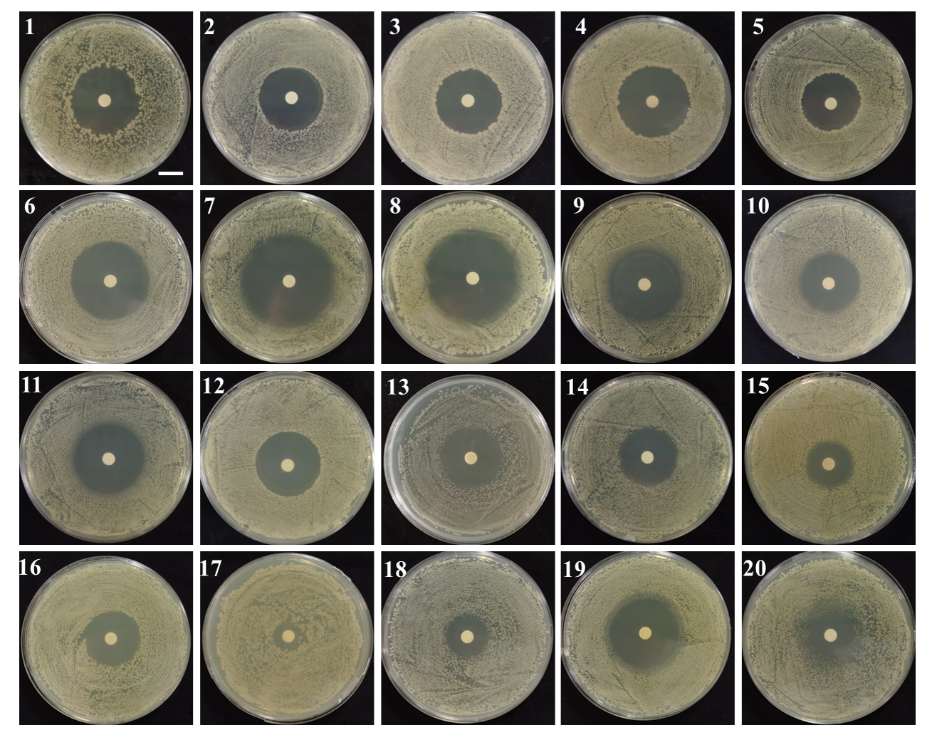
**

**Figure 6. Drug susceptibility testing.** 1，Penicillin; 2，oxacillin; 3，Ampicillin; 4，Carboxybenzyl penicillin; 5，piperacillin; 6，Cephalexin; 7，Cefazolin; 8，Cefradine; 9，Cefuroxime; 10，Ceftazidime; 11，Ceftriaxone; 12，Cefoperazone; 13，Polytetracycline; 14，Amikacin; 15，Gentamicin; 16，Kanamycin; 17, neomycin; 18, Tetracycline; 19, minocycline; 20，Erythromycin. Scalar bar, 10 mm.


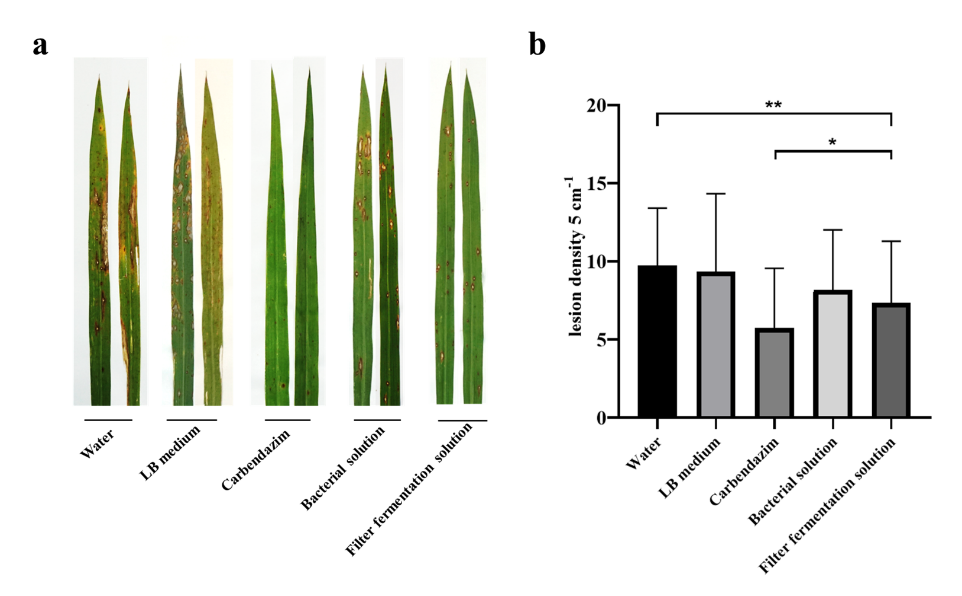


**Figure 7. Disease spots of rice blast Guy11 after 7 days of infection with rice after treatment under different conditions (a). Statistical analysis of the number of disease spots after 7 days (b).** the number of disease spots in 5 cm per leaf was randomly sampled. P<0.05 represents a significant difference.

**
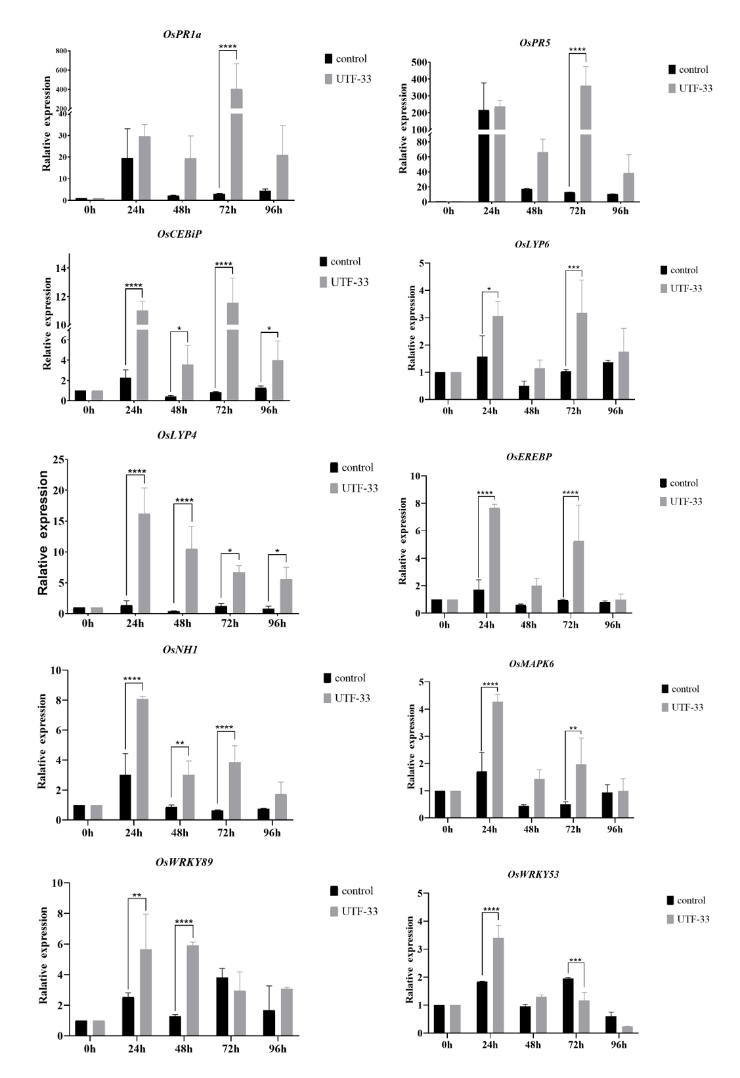
**

**Figure 8. Differences in the expression of antagonistic UTF-33 defense genes in rice.** P<0.05 represents a significant difference.

**
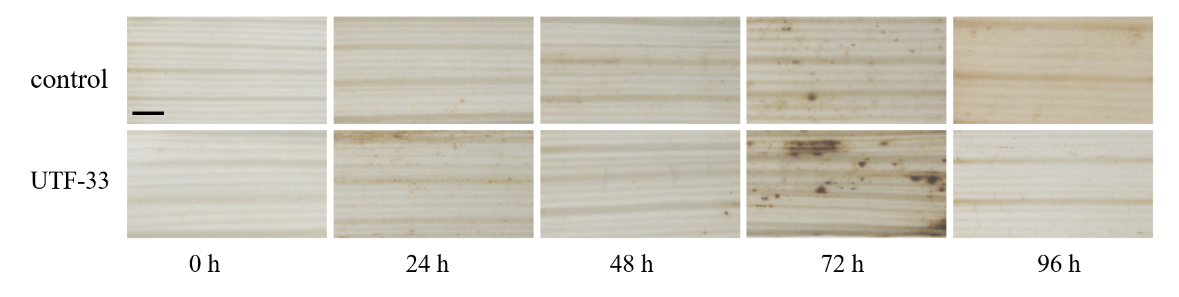
**

**Figure 9. Differences in H_2_O_2_ accumulation arising from antagonistic UTF-33 in rice leaves.** Scale bar, 50 μm.

**
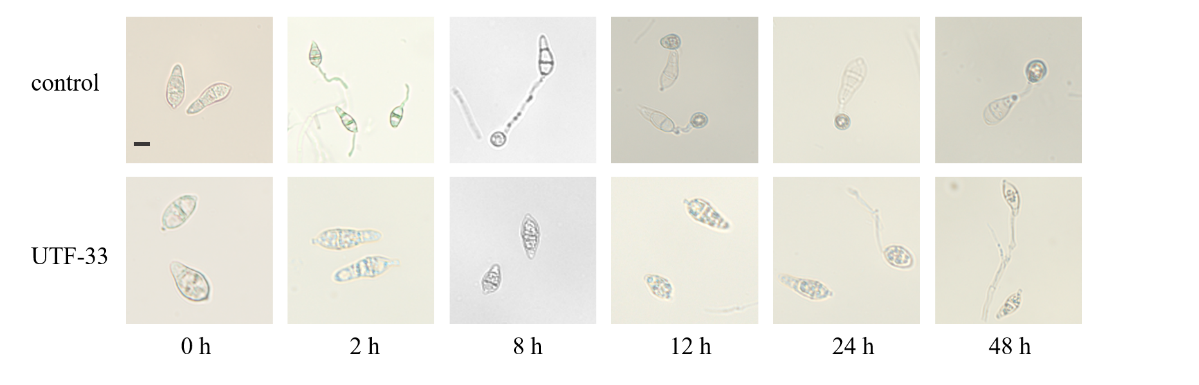
**

**Figure 10. Effect of n-butanol crude extract on conidial germination and the formation of appressorium.** Scale bar, 10 μm.


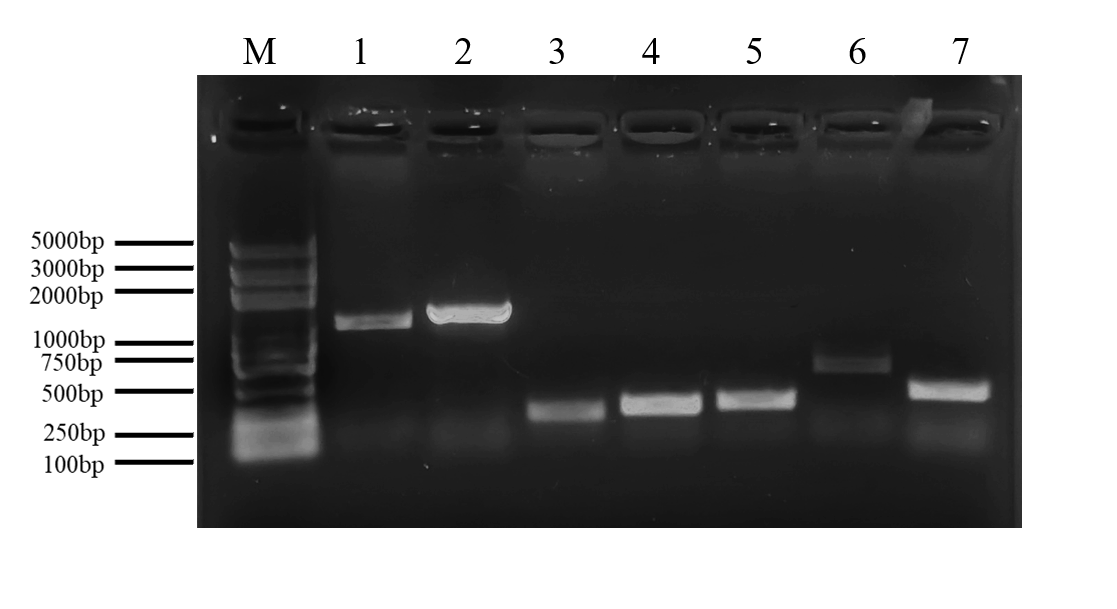


**Figure 11. Electrophoresis analysis of functional gene amplification fragments**


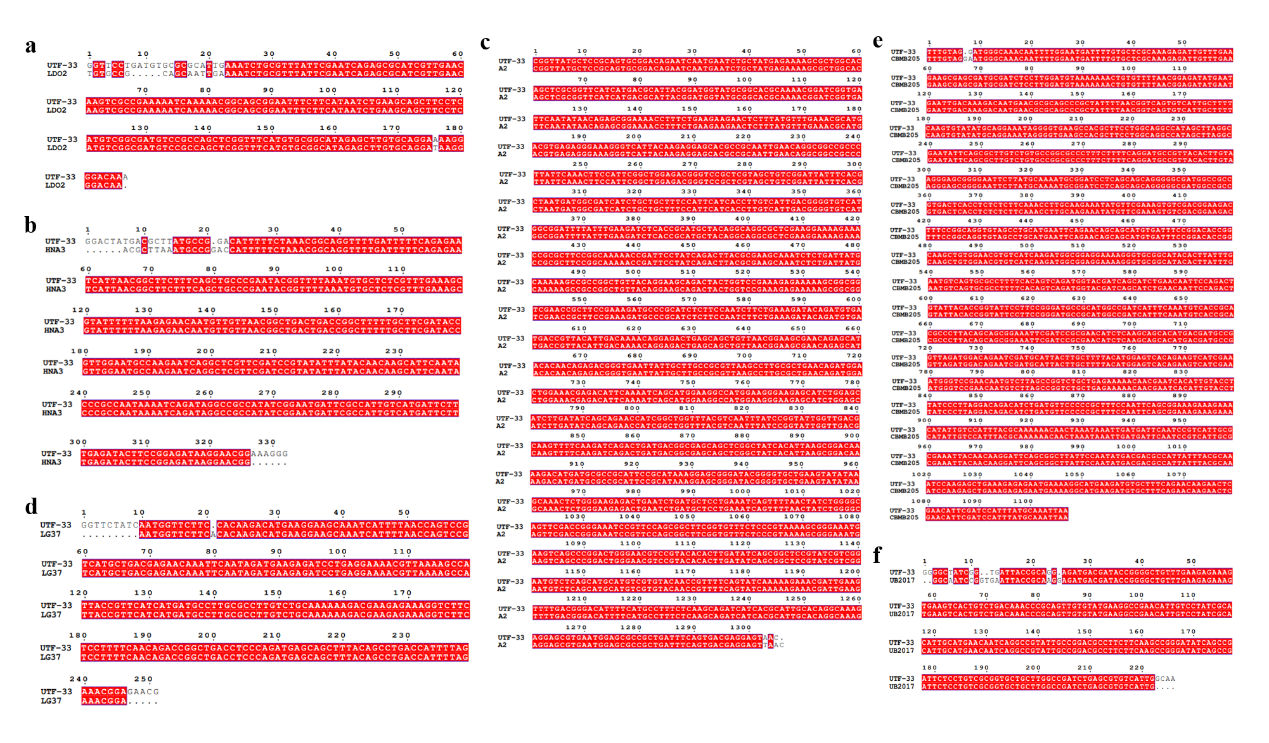


**Figure 12. Nucleic acid sequence comparison plots of functional genes. a-f is corresponding to gene *bioA*, *bmyB*, *fenB*, *fenD*, *ituD* and *srfAA*, respectively.** LDO2: *Bacillus velezensis* LDO2, HNA3: *Bacillus sp*. HNA3, A2: *Bacillus velezensis* A2, LG37: *Bacillus velezensis* LG37, CBMB205: *Bacillus velezensis* CBMB205, UB2017: *Bacillus velezensis* UB2017


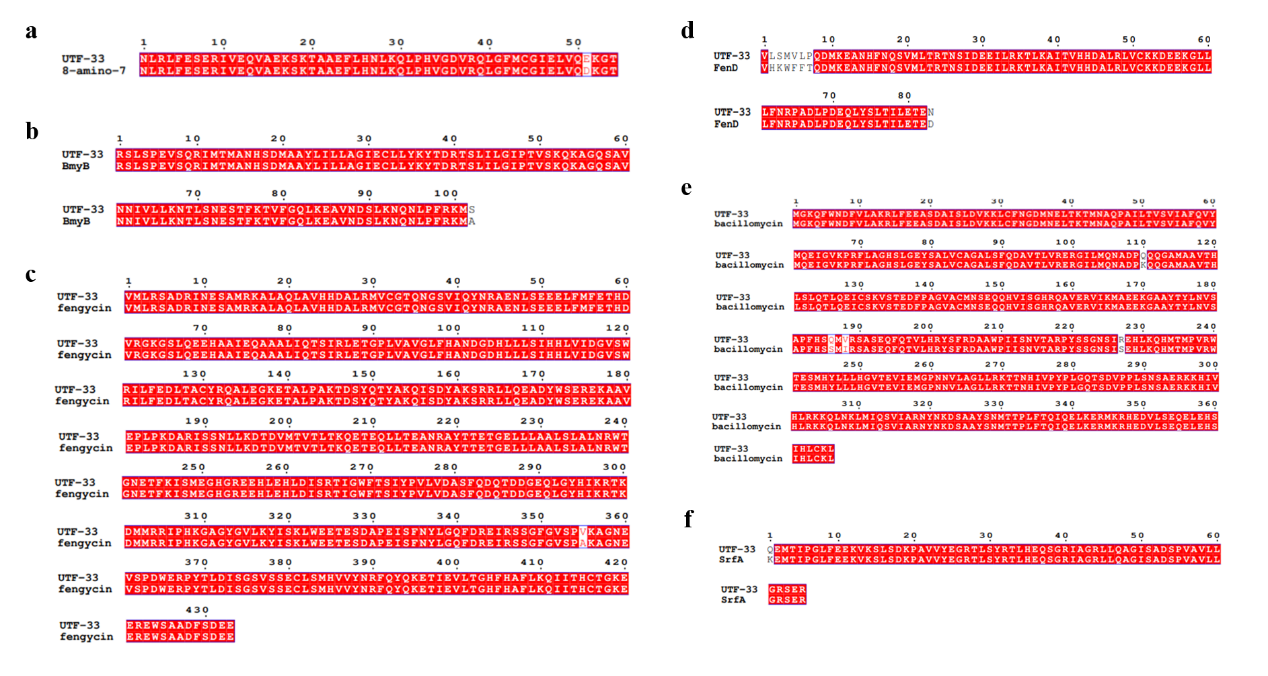


**Figure 13. Comparative amino acid sequences of functional genes. a-f is corresponding to amino acid sequence comparisons of gene *bioA*, *bmyB*, *fenB*, *fenD*, *ituD* and *srfAA*, respectively.** 8-amino-7: adenosylmethionine--8-amino-7-oxononanoate transaminase in *Bacillus velezensis*, BmyB: bmyB in *Bacillus subtilis*, fengycin: fengycin synthetase in *Bacillus subtilis*, FenD: FenD in *Bacillus subtilis*, bacillomycin: bacillomycin D biosynthesis malonyl-CoA transacylase BamD in *Bacillus velezensis*,, SrfA: surfactin non-ribosomal peptide synthetase SrfAA in *Bacillus velezensis*
