# Supplementary figures and images for: Antifungal mechanisms of the antagonistic bacterium Bacillus mojavensis UTF-33 and its potential as a new biopesticide
Source: Front Microbiol. 2023 May 24;14:1201624. doi: 10.3389/fmicb.2023.1201624 (PMC10246745; doi:10.3389/fmicb.2023.1201624)

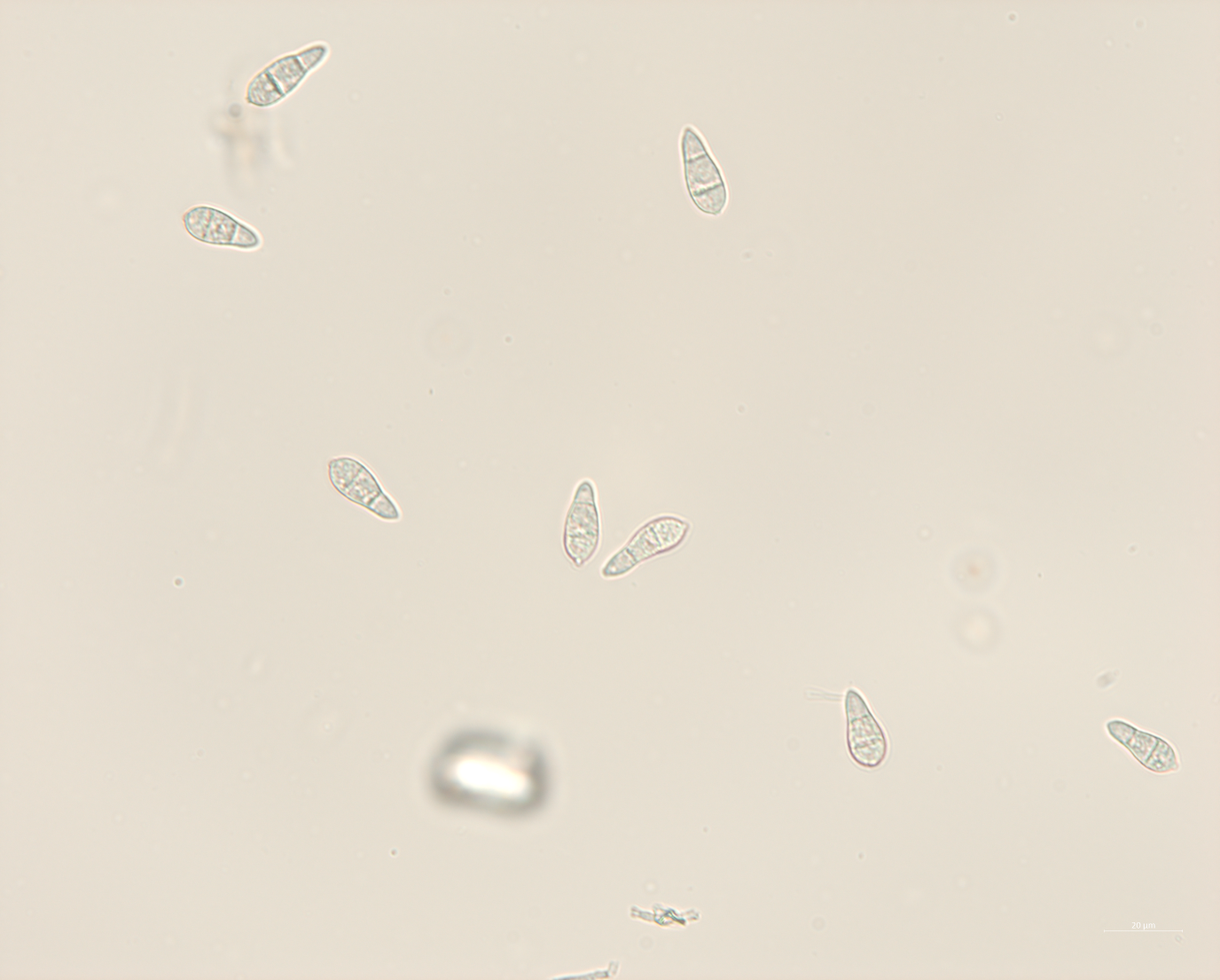

Supplement: Supplementary file 2 [file Image_1.TIF]

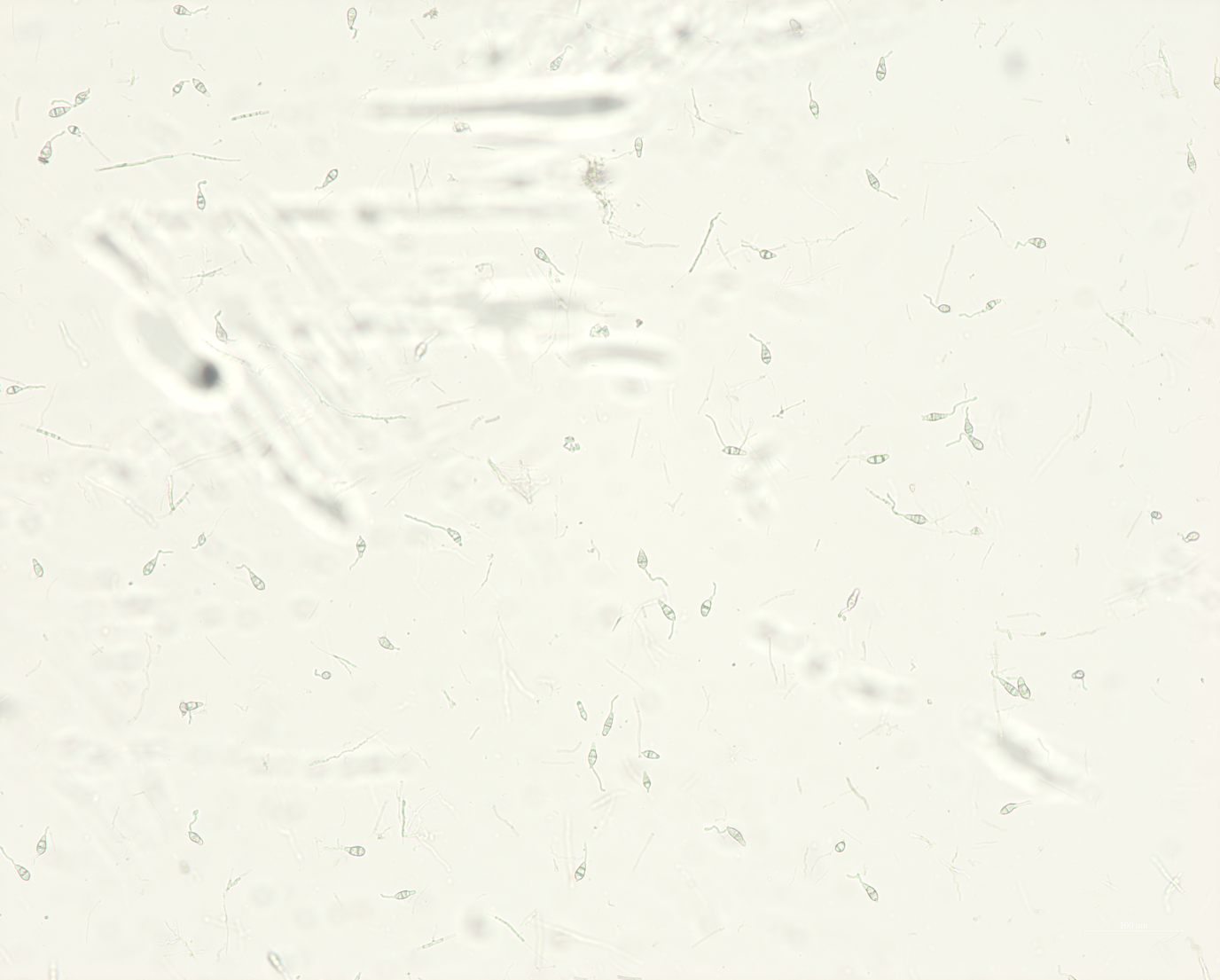

Supplement: Supplementary file 3 [file Image_2.TIF]

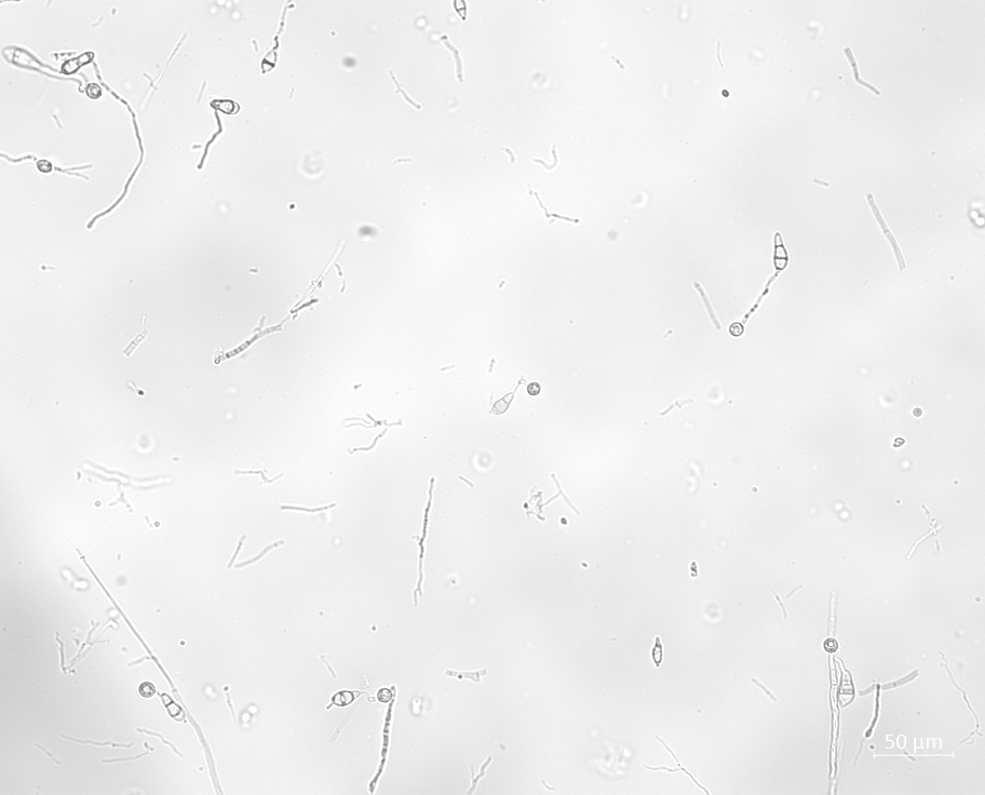

Supplement: Supplementary file 4 [file Image_3.TIF]

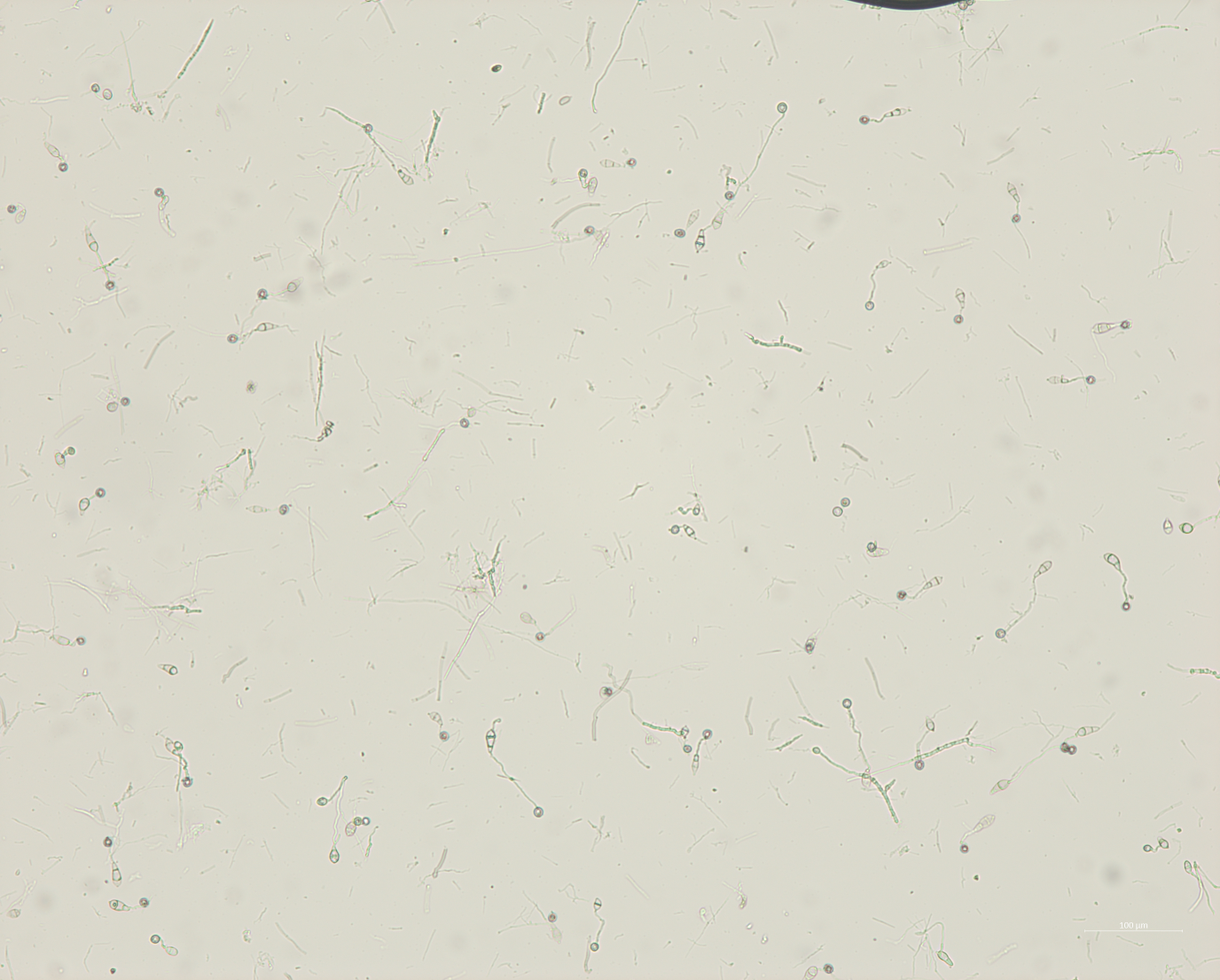

Supplement: Supplementary file 5 [file Image_4.TIF]

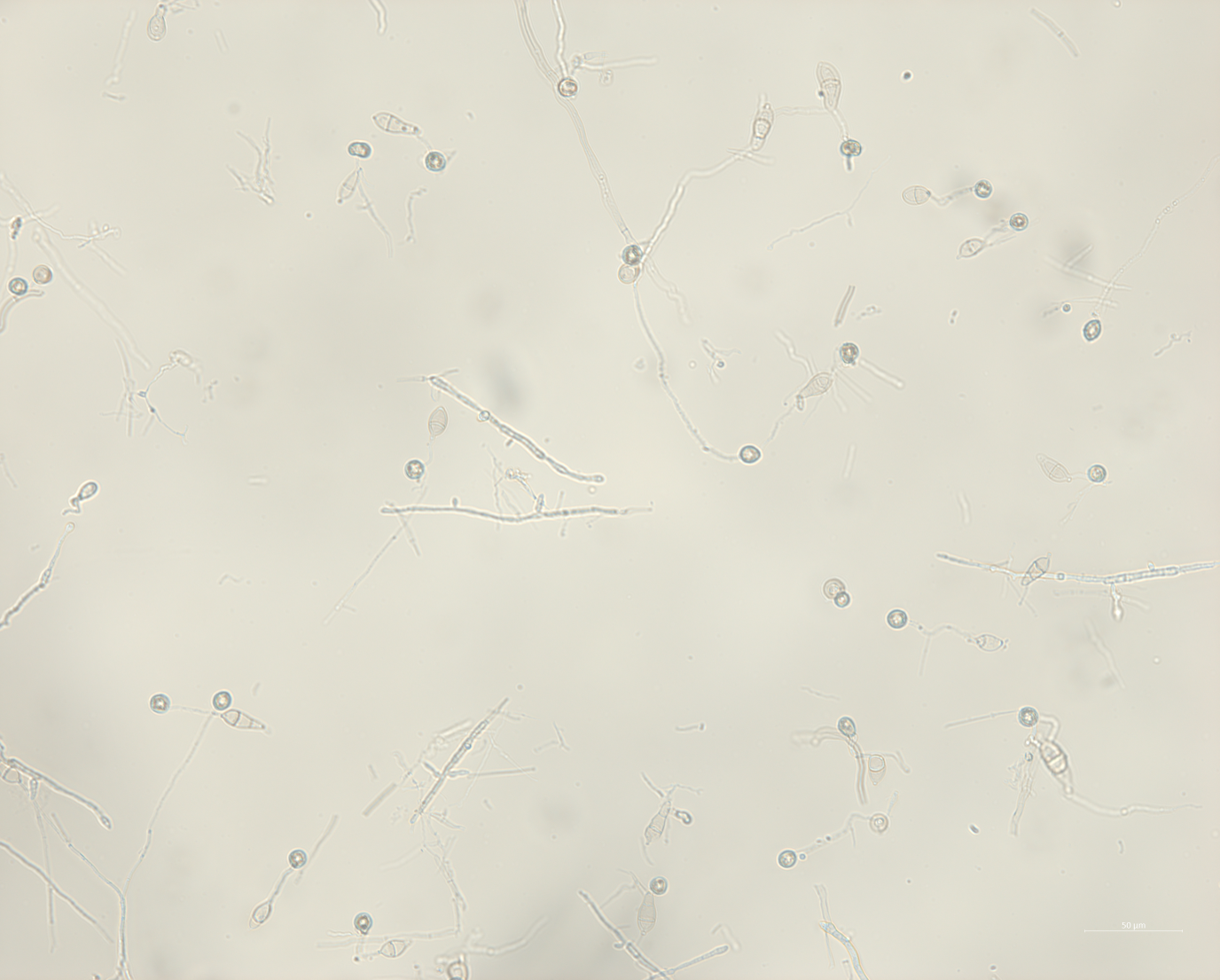

Supplement: Supplementary file 6 [file Image_5.TIF]

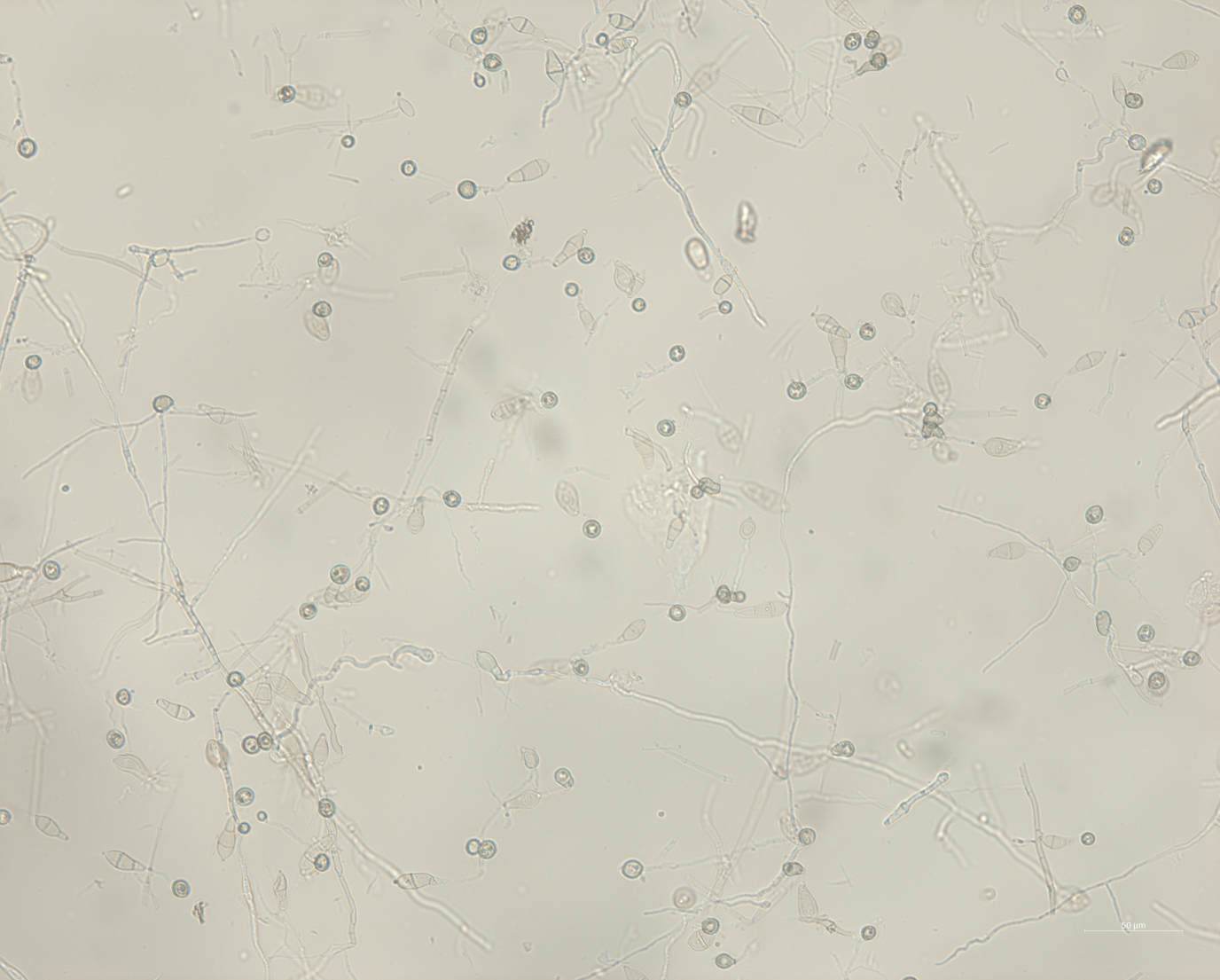

Supplement: Supplementary file 7 [file Image_6.TIF]

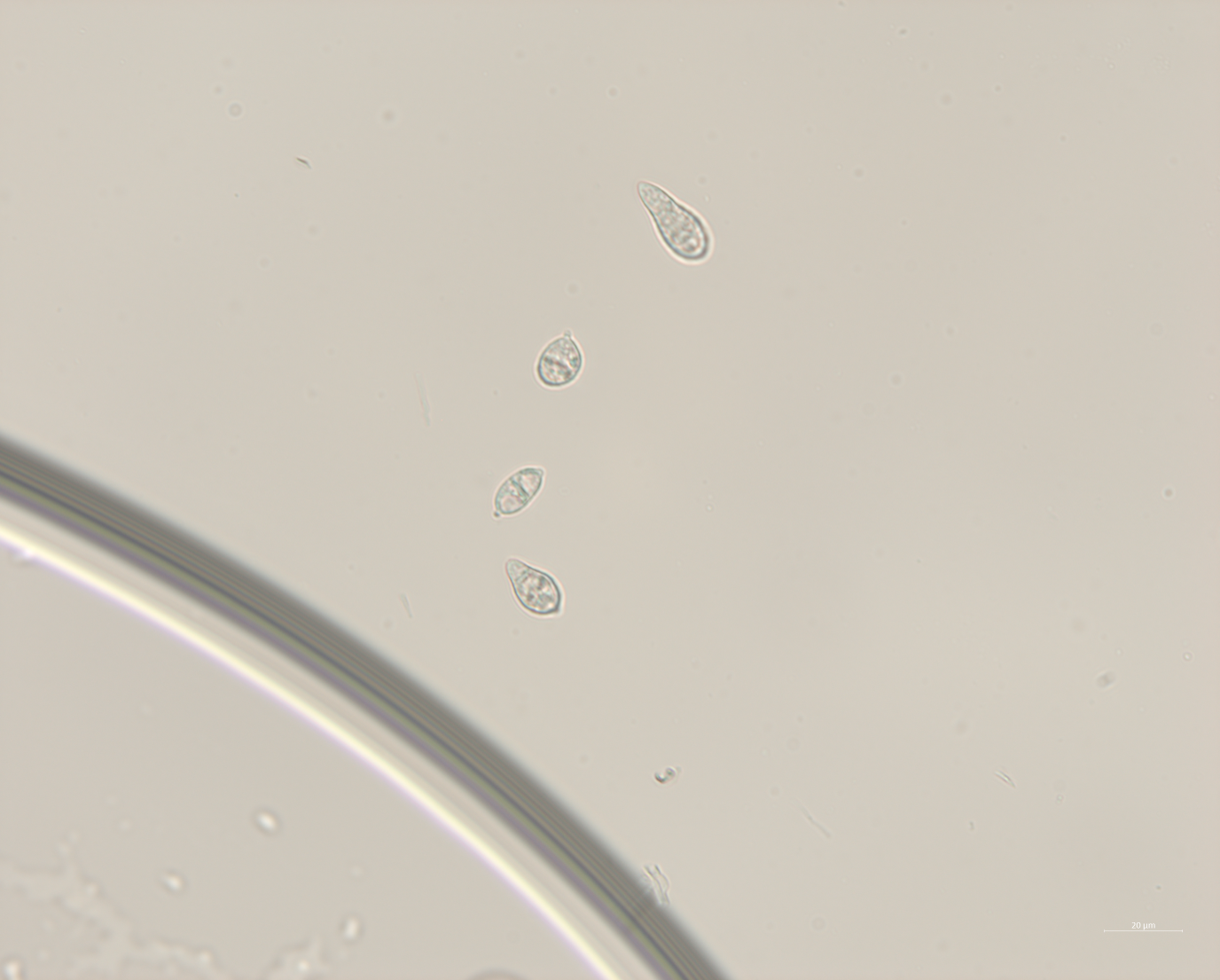

Supplement: Supplementary file 8 [file Image_7.TIF]

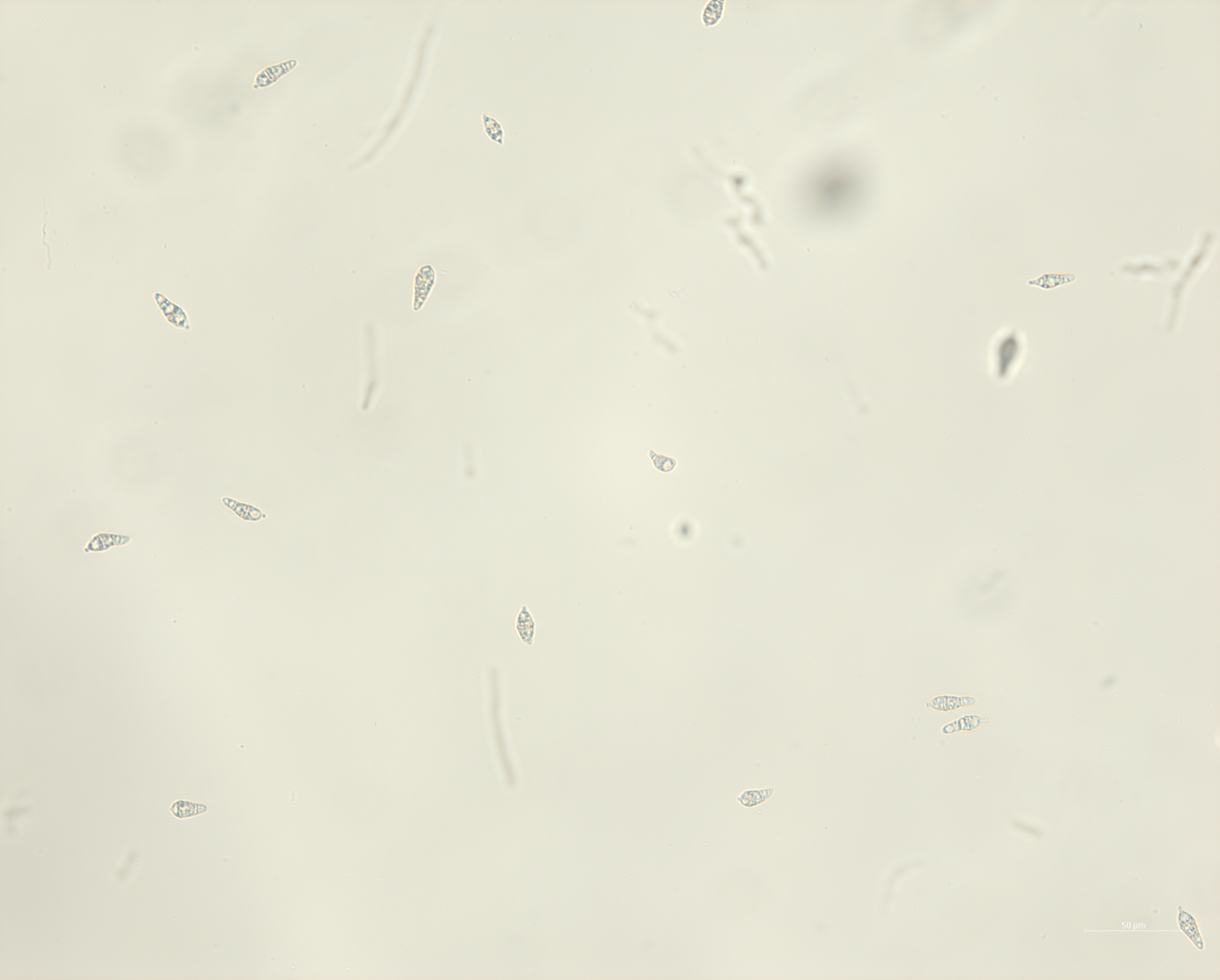

Supplement: Supplementary file 9 [file Image_8.TIF]

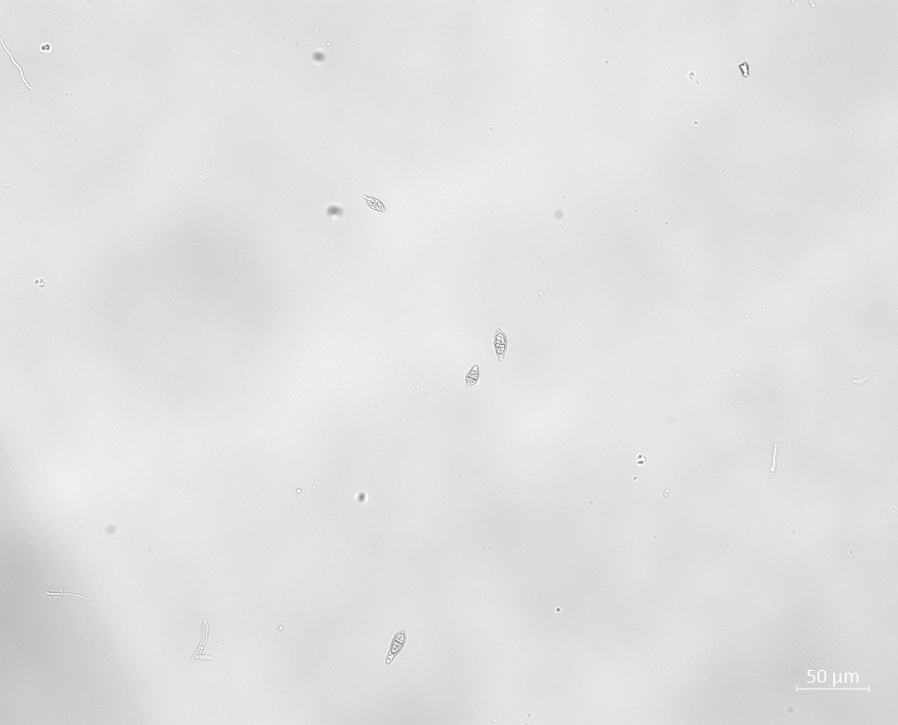

Supplement: Supplementary file 10 [file Image_9.TIF]

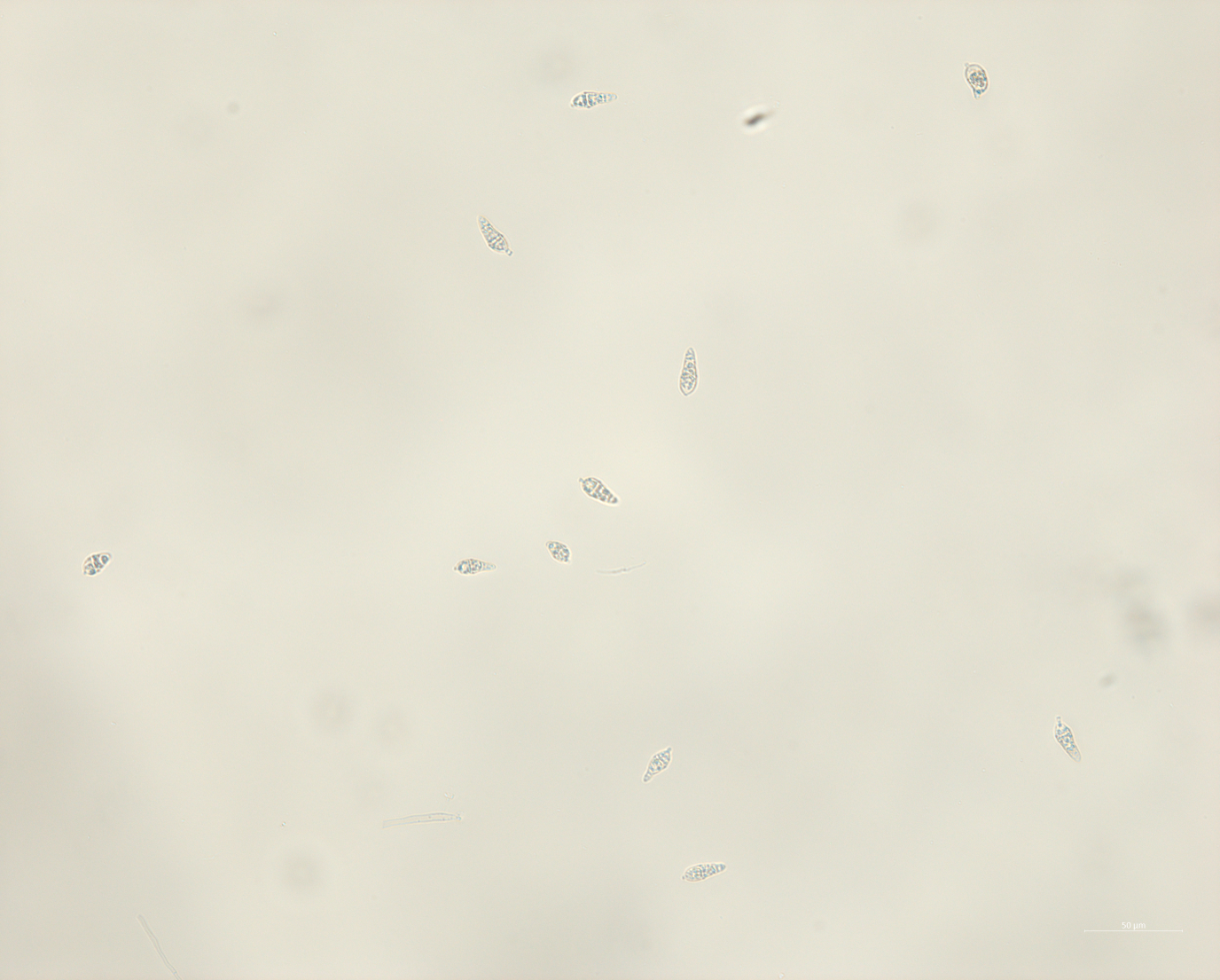

Supplement: Supplementary file 11 [file Image_10.TIF]

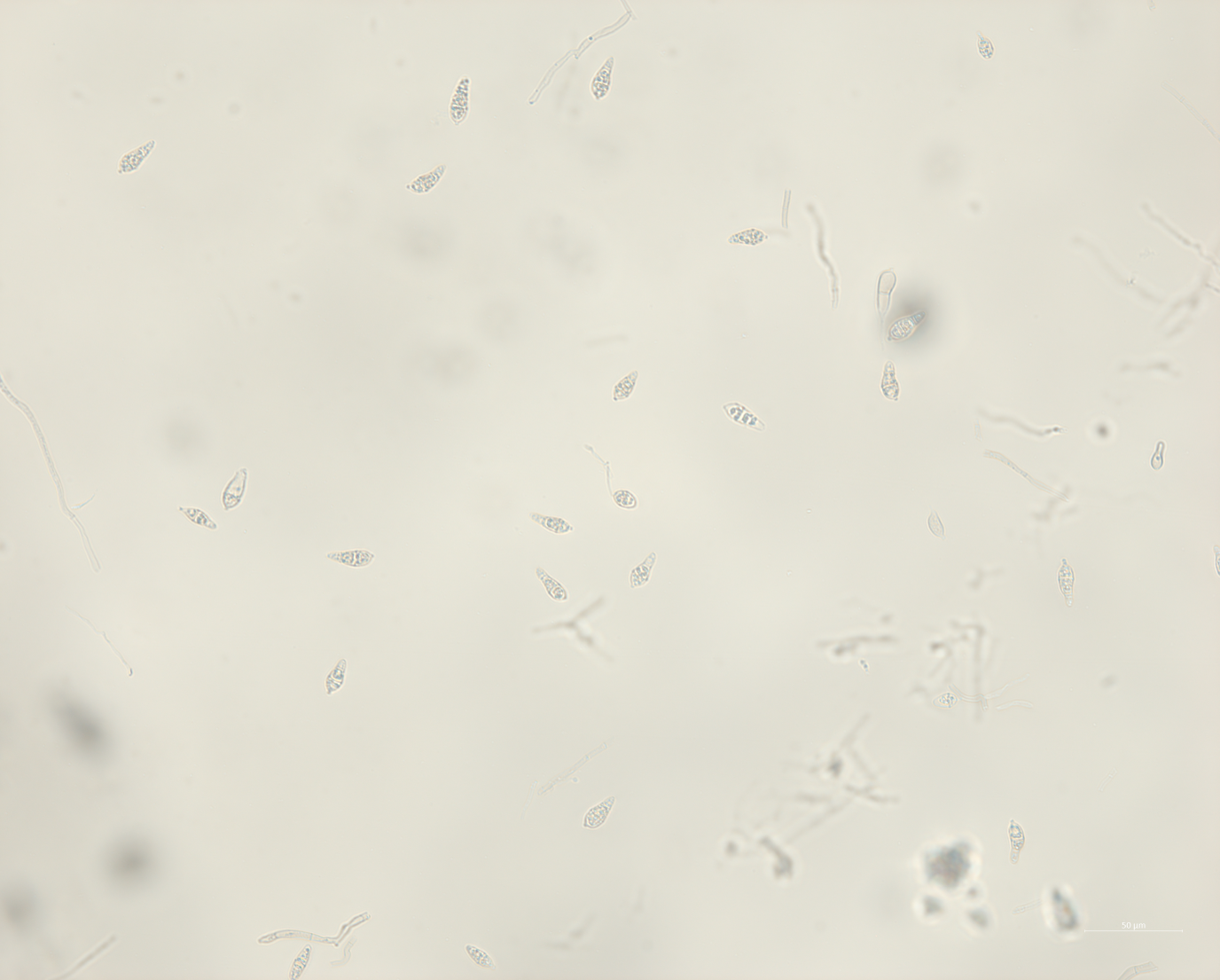

Supplement: Supplementary file 12 [file Image_11.TIF]

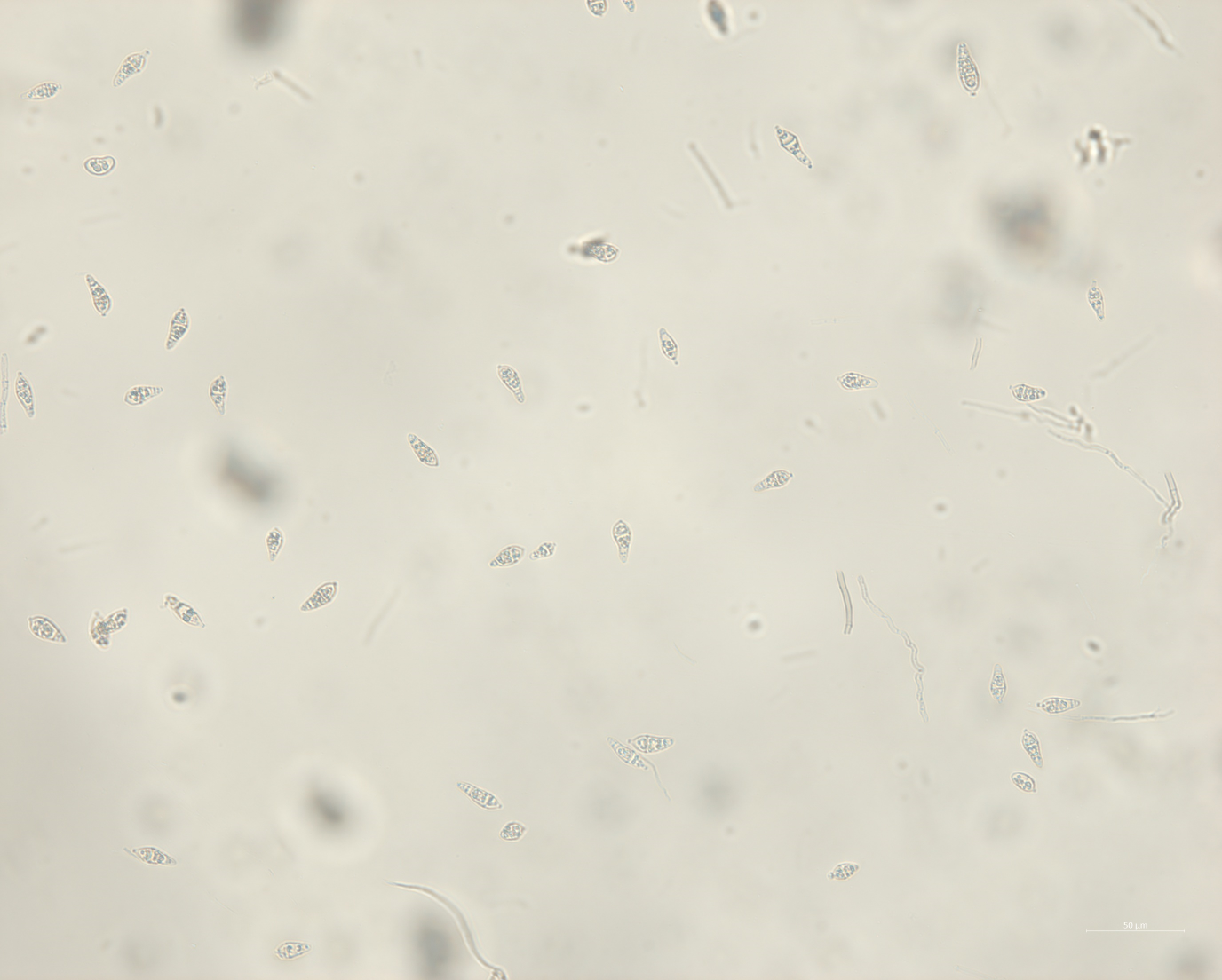

Supplement: Supplementary file 13 [file Image_12.TIF]
