# Supplementary material for: Antifungal mechanisms of the antagonistic bacterium Bacillus mojavensis UTF-33 and its potential as a new biopesticide
Source: Front Microbiol. 2023 May 24;14:1201624. doi: 10.3389/fmicb.2023.1201624 (PMC10246745; doi:10.3389/fmicb.2023.1201624)
Supplement: Supplementary file 14 [file Table_1.DOCX]

| **Table 1 The related defense genes detected in this study and the primers used for fluorescent quantitative PCR** | |
| --- | --- |
| Gene | Primer sequences (5→3′) |
| Actin | F: GAGTATGATGAGTCGGGTCCAG |
|  | R: ACACCAACAATCCCAAACAGAG |
| PR1a | F: GCTACGTGTTTATGCATGTATGG |
|  | R: TCGGATTTATTCTCACCAGCA |
| PR5 | F: GGTACAACGTCGCCATGAGCT |
|  | R: TGGGCAGAAGACGACTCGGTAG |
| CEBiP | F: CATCGCTCATCATACAAACCA |
|  | R: GGAGATAACAGACATGCTCCAC |
| NH1 | F: AAGCGGTTCAAATCTCAAA |
|  | R: GCCTCCATCGGAAACATA |
| MAPK6 | F: CTCGTACCACCTCAGAAAC |
|  | R: AAATACAGCCCACAGACC |
| LYP6 | F: TGCCCAGGACCACATCAGT |
|  | R: CCAGGGAAGCCCGGAATAT |
| LYP4 | F: GCAACTTGGACCTGTTCTGCG |
|  | R: CCTGGGCATTGAGGCTTGAGT |
| WRKY53 | F: ACGGGCAGAAGCAGGTGAAG |
|  | R: CCCTTGTAGACGATCTGGGTGA |
| WRKY89 | F: GCACCTCACAATGATGGA |
|  | R: GGACAGCCTTGCACTTTA |
| EREBP | F: GTGTTCGTGTCTGGCTTGG |
|  | R: CACTTGACTTGGGTGCTTTA |

**Table 2 Primers used for amplification of functional genes relevant to this study**

| Gene |  | Primer sequences (5′→3′) | PCR products size (bp) |
| --- | --- | --- | --- |
| bioA |  | F:TTCCACGGCCATTCCTATAC | 210 |
|  |  | R:TTTGTCCCCTTATCCTGCAC |  |
| srfAA |  | F:GAAAGAGCGGCTGCTGAAAC | 273 |
|  |  | R:CCCAATATTGCCGCAATGAC |  |
| fenD |  | F:CCTGCAGAAGGAGAAGTGAAG | 293 |
|  |  | R:TGCTCATCGTCTTCCGTTTC |  |
| fenB |  | F:CTATAGTTTGTTGACGGCTC | 1400 |
|  |  | R:CAGCACTGGTTCTTGTCGCA |  |
| ituC |  | F:TTCACTTTTGATCTGGCGAT | 575 |
|  |  | R:CGTCCGGTACATTTTCAC |  |
| ituD |  | F:ATGAACAATCTTGCCTTTTTA | 1203 |
|  |  | R:TTATTTTAAAATCCGCAATT |  |
| bmyB |  | F:TGAAACAAAGGCATATGCTC | 395 |
|  |  | R:AAAAATGCATCTGCCGTTCC |  |
